# Breast Cancer Multicellular Spheroid Models—A Tool for Studying Cancer Biology; a Possible Platform for Drug Screening and Personalized Medicine

**DOI:** 10.3390/ijms27031314

**Published:** 2026-01-28

**Authors:** Maksymilian Kolodziej, Jakub Czarny, Hanna Dyla, Dominika Rekawek, Maria Zlotek, Alicja Wlodarz, Michal Baranowicz, Eliza Kwiatkowska-Borowczyk, Olga Milbrandt, Rodryg Ramlau, Hanna Dams-Kozlowska

**Affiliations:** 1 Student Scientific Society, Poznan University of Medical Sciences, 61-701 Poznan, Poland; 86067@student.ump.edu.pl (M.K.); 86156@student.ump.edu.pl (J.C.); 86185@student.ump.edu.pl (M.B.); 2 Faculty of Medicine, Collegium Medicum, The University of Opole, 45-040 Opole, Poland; 132841@student.uni.opole.pl (H.D.); 132842@student.uni.opole.pl (D.R.); 132189@student.uni.opole.pl (M.Z.); 131950@student.uni.opole.pl (A.W.); 3 Department of Diagnostics and Cancer Immunology, Greater Poland Cancer Centre, 61-866 Poznan, Poland; eliza.kwiatkowska@wco.pl; 4 Oncology Clinic, Institute of Oncology, Poznan University of Medical Sciences, 61-701 Poznan, Poland; omilbrandt@ump.edu.pl (O.M.); rodrygramlau@ump.edu.pl (R.R.); 5 Department of Cancer Immunology, Poznan University of Medical Sciences, 61-701 Poznan, Poland

**Keywords:** breast cancer, multicellular spheroids, personalized medicine, preclinical drug development

## Abstract

There is a growing pressure for further breast cancer treatment development to improve patient quality of life and clinical effectiveness while reducing drug resistance and side effects. Clinical research, however, remains both expensive and time-consuming, highlighting the importance of advancing preclinical studies. The ability of a model to accurately replicate the biological environment is essential for generating reliable data. Although research on two-dimensional in vitro cell culture systems has been conducted for over a century, their limited complexity hinders further development of cancer treatments. Consequently, the response to tested medications is artificial, since some drugs appear to be effective only in the two-dimensional model and not in clinical settings. A multicellular spheroid (MCS) is a three-dimensional assembly of cancer cells, used in oncology to study cancer biology and drug sensitivity. MCSs serve as valuable in vitro models in preclinical drug development. They can also be used in a clinical setting as a personalized tool for drug susceptibility screening. The objective of this review is to highlight new possibilities for improved anticancer drug screening and the development of personalized treatment regimens with multicellular spheroids.

## 1. Introduction

Breast carcinoma is a significant health issue that urgently needs to be addressed. Treatment options for breast cancer include surgical procedures, radiation, hormone therapy, chemotherapy, and novel therapies [1]. However, despite advances in oncological research, drug resistance still plays a significant role in therapy failures [2]. Drug testing and development rely on generating models that closely mirror the environment of the human body and in vivo cancer behavior. Chemotherapeutic drugs were initially tested on animals [3], a method still in use [4]. Nowadays, however, concerns have arisen about animal well-being and the suitability of these models [5]. Two-dimensional (2D) monolayer cell cultures are also used for drug testing; however, they represent an unnatural environment, lacking cell–cell and extracellular matrix (ECM) interactions, polarity, and physiological gradients (such as nutrients and oxygen), which are necessary to study drug responses. These properties can be provided by three-dimensional (3D) conditions, for example, on multicellular spheroids (MCS)—a 3D cell aggregate [6]. The studies on 3D models revealed novel drivers crucial for cancer growth [7] and have contributed to the development of radiotherapy [8].

It is generally well accepted that three-dimensional cultures can better simulate the complex cell-to-cell interactions present within a tumor than two-dimensional cell cultures [9,10]. Additionally, 3D models more accurately reflect the natural behavior of cancer. For example, experimental evidence indicates that 3D cancer cell cultures can mimic physical barriers or hypoxia observed within tumors; the factors that significantly contribute to drug resistance due to limiting drug penetration [11]. The Gong et al. group tested cytotoxicity of doxorubicin on MCSs derived from MCF-7 breast cancer cells, showing that the drug signal was restricted to the outer spheroid layer, with no detectable signal in the core [12]. Another group, Horning et al., revealed differences in anticancer drug resistance by studying the MCF7 breast cancer cell line [13]. Drugs, such as doxorubicin, paclitaxel, and tamoxifen, showed significant efficacy in 2D cultures but exhibited altered responses in more complex 3D models, indicating that the high collagen content in 3D models acted as a barrier to drug diffusion [13]. Hypoxia is not observed in monolayered 2D cell cultures, whereas in 3D models, it can be generated within cell masses due to limited oxygen/nutrient diffusion gradients [14,15,16,17]. Through altered signal transduction, histone acetylation, gene expression, and protein expression, hypoxia affects many cellular functions, including metabolic adaptation, angiogenesis, migration, invasion, stromal modeling, and drug resistance [14]. To summarize, 3D models more accurately reflect natural cancer behavior, potentially contributing to gaining more valuable insights into drug responses [18,19].

However, the precise in vitro prediction of in vivo tumor cell responses to treatment is challenging. On one side, MCSs serve as more suitable models for drug screening due to their structural similarities to in vivo tumors [20]. Nevertheless, an MCS based on the culture of only the cancer cells (monoculture, homospheroids) remains insufficient as a 3D model for evaluating the efficacy of anticancer therapy, primarily because they lack all components of the tumor microenvironment (TME) [21]. It is essential to understand a tumor as a complex structure composed of multiple interacting cell types, rather than just a single population of cancer cells. A special role is attributed to interactions between tumor cells and non-cancerous cells, collectively defined as the TME [22,23,24,25,26,27,28]. The TME consists of fibroblasts, immune cells (including macrophages, T cells, B cells, and NK cells), endothelial cells, and lipocytes. It also includes the extracellular matrix (ECM) and many signaling molecules [26,27,29,30]. Thus, it became necessary to develop new models that incorporate additional cellular and structural components of the tumor. Heterotypic tumor spheroids are a model for studying interactions within the TME, as well as tumor responses to anticancer treatment [31]. Heterotypic models exhibit reduced sensitivity to compounds of anticancer therapy compared to homotypic spheroids [28]. For example, in breast cancer, it was observed that spheroid co-culture with stromal and endothelial cells exhibits reduced sensitivity to lapatinib compared with homotypic BT-474 cancer spheroids [32].

Among the components of the TME, breast cancer stem cells (BCSCs) are of special interest. BCSCs represent an important subpopulation of cancer cells [33]. They play a crucial role in the physiological and biological processes that drive breast tumor development and progression, such as recurrence, vasculogenic mimicry, angiogenesis, metastasis, and anticancer drug resistance [34]. Insensitivity to conventional treatment regimens, including chemotherapy and hormone therapy, is associated with properties of BCSCs [35,36]. Consequently, therapies targeting BCSCs represent a promising strategy for breast cancer treatment. Thus, many in vitro methods have been developed to treat BCSCs, and the MCS model constitutes an important tool in drug screening for these difficult-to-target cells.

Finally, personalized medicine is a primary goal for the future of breast cancer treatment [37]. It is important to recognize that breast cancer is not a single disease, but a collective term for multiple distinct conditions. Each subtype of breast cancer differs in the biological and molecular characteristics and hormone receptor status and requires a different therapeutic approach [38]. Nowadays, personalized anticancer treatment, dedicated to an individual’s unique cancer characteristics, is crucial for achieving optimal outcomes. Therefore, matching a patient’s tumor-specific characteristics to the most suitable therapy is crucial. To accomplish this goal, patient-derived models (such as patient-derived xenograft (PDX), patient-derived organoids (PDOs), patient-derived spheroids (PDSs)) can provide a promising approach for identifying the most appropriate therapy [39]. Standardization of PDS culture protocols is essential to achieve faster results than with PDOs and PDXs. Capturing the specific profile of a patient’s tumor could significantly improve clinical outcomes.

The scope of this review is to identify new opportunities to improve anticancer drug screening in breast cancer. A comprehensive understanding of the applications and limitations of available experimental models is crucial for better interpreting preclinical findings and then improving clinical outcomes. Drug resistance is one of the significant causes of treatment failure. Thus, the development of in vitro methods that properly mimic cancer in vivo should contribute significantly to the progress of breast cancer treatment and beyond. The majority of studies focus on a limited number of models within a single article. This review fills this gap by offering an integrated summary and comparison of the currently used experimental models, emphasizing how each contributes to understanding breast cancer biology and therapeutic response. We reviewed various breast cancer MCSs, including homotypic, TME-mimicking heterotypic types, with a special highlight of the importance of BCSC-based spheroids, and finally focused on the development of personalized treatment regimens using various forms of patient-derived models, including multicellular spheroids. Although patient-derived xenografts are widely regarded as one of the best preclinical drug screening models available, their disadvantages remain, particularly the cost and time required to produce them. PDSs might become an important tool if the standardization protocols are established, as they may lower the cost of experiments and expedite the process.

We believe that this review will bring attention to both preclinical scientists and clinicians to new patient-derived methods, especially multicellular spheroids, and their potential future applications in personalized medicine and drug screening.

## 2. Important Factors for Developing In Vitro Cancer Models

### 2.1. Interactions Among the Components of the TME and Their Significance in Potential Anticancer Therapies

**Figure 1:**
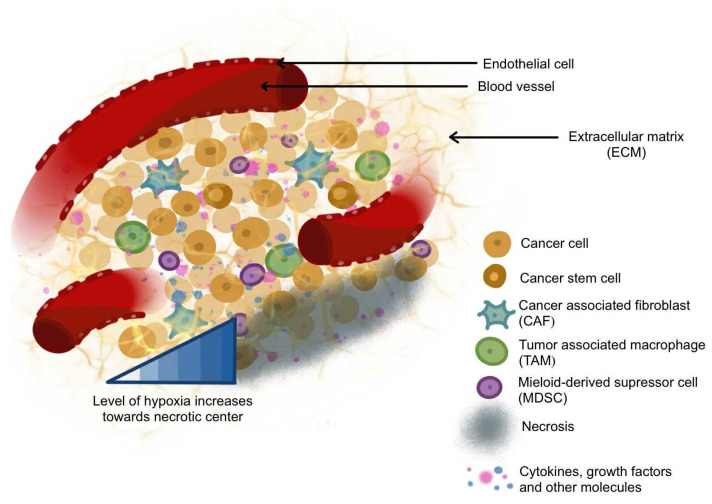
A scheme of a tumor microenvironment, indicating a complex system of various tumor, stromal, and immune cells. The 3D model mimicking the TME in an in vitro setting may enable the study of the tumor microenvironment and its effects on drug behavior, which is crucial to the drug development process.

As mentioned above, the TME refers to the complex system of various components, including non-cancerous cells, blood vessels, soluble molecules, and the extracellular matrix, which actively support not only cancer growth, survival, and invasion but also resistance to therapy (Figure 1). Using the example of breast cancer, it can be stated that cellular interactions within the TME are organized hierarchically, with specific cell types exerting greater influence. At the top of this hierarchy are cancer-associated fibroblasts (CAFs), followed by components of TIME, especially tumor-associated macrophages (TAMs) [28,40]. However, it should be noted that in the TME, interactions are multidirectional; non-cancerous cells interact not only with cancer cells but also with each other and the ECM. These multidirectional interactions are responsible for cellular plasticity, directly influencing the development of drug resistance.

Although certain CAF subpopulations, such as cancer-restraining CAFs, inhibit tumor progression [41], most subpopulations of CAFs promote cancer development [42,43,44]. Among other processes, CAFs are known to promote drug resistance and cancer relapse through several mechanisms, including the induction of epithelial–mesenchymal transition (EMT), activation of stemness pathways, remodeling of the ECM, and dysregulation of cellular metabolism [45]. For example, in breast cancer, CAFs are responsible for tamoxifen resistance by activating PI3K/AKT (Phosphoinositide 3-kinase/Protein Kinase B) and/or Ras/Raf/MEK1/ERK1/2 (a Family of Small GTPases/a Serine/Threonine Protein Kinase/Mitogen-Activated Protein Kinase/Extracellular Signal-Regulated Kinase) pathways, which is accompanied by hyperphosphorylation of ERα (Estrogen Receptor Alpha) at Serine 118 [46,47].

Additionally, CAFs play an important role in the tumor pathophysiology, especially regarding interactions with TIME components [48,49,50]. Through these interactions, CAFs exert an immunosuppressive effect, such as reducing migration of CD8(+) T cells to juxtatumoral stromal compartments and preventing their access to cancer cells by secreting different chemokines [51]. In addition, CAFs are responsible for recruiting monocytes and promoting their polarization into the M2 phenotype of TAMs, which are pro-tumorigenic macrophages [52]. In breast cancer, for example, CAFs promote the above-mentioned processes by secreting monocyte chemotactic protein-1, SDF-1, and chitinase 3-like 1 [53,54].

Among TIME components, TAMs are the most significant, as they have a crucial impact on modulating tumors, particularly in terms of tumor immune suppression [47,55]. TAM cells have been shown to promote growth, metastasis, and drug resistance through various mechanisms, including metabolic support (via exosome secretion), immunosuppression (TGF-β, IL-10), ECM remodeling (MMP), angiogenesis (VEGF), promotion of epithelial–mesenchymal transition, support of cancer stem cells, enhancement of drug efflux, and regulation of drug metabolism [56]. For instance, TAMs promoted paclitaxel resistance in MDA-MB-231 breast cancer cells through the activity of cathepsin proteases [57].

Although CAFs and TAMs are considered the most significant factors in the TME, numerous other components, including endothelial cells, adipocytes, stellate cells, ECMs, and exosomes, also influence tumor pathophysiology and drug resistance [58]. Neutrophils are a notable example, which, upon activation, form neutrophil extracellular traps (NETs) [59]. NETs cannot only be responsible for cancer initiation [60,61,62] but also can contribute to therapy resistance. Targeting NETs with conventional immune therapies has been shown to reduce drug resistance and enhance the efficacy of anticancer treatments [63]. For example, the application of eukaryotic deoxyribonuclease I (DNase I) may enhance the efficacy of CAR-T therapy by mitigating the impact of NETs on the TME [64].

The ECM is a significant component of the TME, comprising collagen, fibronectin, elastin, and laminin. Many elements of the TME, especially CAFs, are responsible for producing ECM components [58]. The ECM not only provides structural support but also supplies growth factors, which are essential for the survival of cancer cells. Matrix metalloproteinases (MMPs) mediate the release of these growth factors through ECM degradation [16]. For example, by releasing MMP-13, the ECM provides an important source of VEGF, a key factor in angiogenesis [58]. The ECM facilitates not only cell adhesion and communication but also guides essential cellular functions such as growth, differentiation, and migration. The ECM is capable of restricting the early-stage progression of cancer [24].

Due to the importance of TME interactions, modulating the TME represents a new approach to cancer treatment. In particular, targeting CAFs to enhance the anticancer immune response is a promising therapeutic strategy, including direct CAF depletion, inhibition of signaling pathways, and functional reprogramming, along with the restriction of CAF-induced ECM remodeling [16]. CAF-specific therapies may complement conventional immunotherapies, such as immune checkpoint inhibitors, including ipilimumab and nivolumab [65]. Moreover, several strategies have been developed to overcome TAM activity in the TME, including depletion of TAMs, repolarization toward the anti-tumor M1-like phenotype, or blocking of monocyte recruitment [56].

As indicated above, the TME is a complex and diverse community, and its components collectively shape tumor-related processes. Understanding these complex interactions is crucial for comprehending the pathophysiology of the tumor and the mechanisms involved in the development of drug resistance. Suitable in vitro models would be helpful in accurately examining the interactions among TME components and processes that occur within. Since 2D cell culture conditions do not provide spatial cellular interactions, only 3D in vitro models can be in focus. Although homotypic 3D cell culture has already provided initial data, mainly on cancer biology, a growing body of evidence suggests that heterotypic cultures are the next step in developing in vitro cancer models for cancer biology and drug screening.

### 2.2. Breast Cancer Stem Cells and Their Significance in Potential Anticancer Therapies

Breast tumor masses comprise diverse cancer cell populations, and breast cancer stem cells (BCSCs) represent only a small subset of them [33]. Although BCSCs comprise a small fraction of the tumor, they are a significant component of the TME. They are recognized as major contributors to breast cancer tumorigenesis, metastasis, drug resistance, and tumor recurrence [66].

BCSCs were first identified by Al-Hajj et al. [67]. Their study revealed that a small tumorigenic population can generate the phenotypic heterogeneity found in the initial tumor [67]. BCSCs are commonly marked by CD44^high^/CD24^low^ or aldehyde dehydrogenase-positive (ALDH^+^) phenotypes [67,68]. The origin of BCSCs remains unclear. One hypothesis suggested that CSCs arise from abnormally regulated stem cells, which leads to self-renewal and differentiation capacity [69,70,71]. The second hypothesis states that epithelial–mesenchymal transition (EMT) is responsible for the development of BCSCs [72]. Both stem cells and CSCs share properties with cells that have undergone EMT [72]. BCSCs are capable of self-renewal and differentiation [73,74]. Many signaling pathways are deregulated in BCSCs, including Notch, Hedgehog, Wnt, and Hippo pathways [75,76,77,78,79,80,81,82,83,84,85]. Several studies emphasize the significant effect of these pathways on tumor resistance, recurrence, and metastasis [75,76,77,78,79,80,81,82,83,84,85]. It is reported that eliminating 50% of BCSCs decreases tumor progression; however, the tumor eventually recovers as long as a stem cell remains [86].

Given the crucial role of BCSCs in tumor development and recurrence, furthering our understanding of the biology of these cells is a necessary condition for the development of more effective anticancer therapies. Based on current knowledge, BCSCs may be a key factor in combating cancer drug resistance. Thus, in vitro research in 3D models may constitute an important step toward achieving this goal, and heterotypic 3D cancer models that incorporate CSCs would be an interesting alternative.

## 3. Anticancer Drugs Tested in Breast Cancer Spheroids

As mentioned above, drug resistance is a significant problem in cancer therapy and significantly contributes to treatment failures. Many efforts are underway to understand the biology of cell resistance to drugs and to conduct studies that would allow us to understand cell sensitivity to a given drug. Obtaining such data would enable the use of the most effective drug, which would have a significant impact on the treatment process and the patient’s health. Awareness of these interdependencies has long been recognized, but progress in developing methods that effectively select effective drugs remains insufficient. Initially, 2D cell culture systems were used for these purposes, but numerous studies now indicate that 3D cell cultures are a potentially superior tool for drug testing. As mentioned earlier, 3D cultures not only allow for cell interactions but also reproduce some of the characteristics of cancer (such as hypoxia and physical barriers). Many studies have indicated that the IC50 (half-maximal inhibitory concentration) of anticancer drugs tested in 3D spheroids is higher than that observed in 2D monolayer models. This difference can be attributed to enhanced cell adhesion interactions and increased levels of MDR-1 (multidrug resistance protein 1), as observed in breast cancer MCF-7 and MDA-MB-231 breast cancer spheroids [87,88]. However, other factors are also in the scope, as the IC50 value has some limitations, including high variability depending on the test conditions, the chosen experimental endpoint, or the method used to assess cell viability. Nowadays, new evidence indicates that the model of 3D spheroids based only on cancer cells (homotype) can also be an insufficient method for drug testing proposals. The heterotype 3D spheroids, which comprise cancerous and non-cancerous cells, the ECM, and active molecules, can more precisely mimic the tumor interactions. Non-cancerous components can actively influence cancer drug resistance. Additionally, the role of BCSCs in the development of drug resistance in cancer has been identified as crucial. Considering the importance of all the factors mentioned above, we have provided numerous examples of reports testing drugs in various in vitro spheroid systems, categorized into homo- and hetero-spheroids and those involving BCSC-based spheroids. A systematic review allows us to see the efficacy of drugs and the relationship between their effectiveness and the type of in vitro cell culture.

### 3.1. Application of Homotypic 3D Breast Cancer Spheroid for Testing Drug Activity

Although the homotypic 3D spheroid models are the simplest of the 3D cell culturing methods, and studies conducted on these models have provided significant information not only on the biology of breast cancer but also on the sensitivity of cells to drugs and the barriers associated with drug therapy.

#### 3.1.1. Analysis of Cell Sensitivity to Conventional Chemotherapeutics on 3D Homotypic Breast Cancer Spheroid Models

A significant portion of research is conducted on three phenotypically distinct breast cancer cell lines, MDA-MB-231, SK-BR-3, and MCF-7, which are commonly categorized as “triple negative B,” “HER2+,” and “luminal A,” respectively, and which exhibit different aggressive properties, with the former being the most aggressive and the latter the least aggressive. Kessel et al. examined the effects of doxorubicin, paclitaxel, 8-quinolinol, and salinomycin on homotypic spheroids derived from MDA-MB-231, SKBR-3, MCF-7, and MDA-MB-436 cell lines based on sphere formation, morphology, and viability analysis [89]. The drug treatments induced various dose-dependent effects, where doxorubicin demonstrated the highest cytotoxicity. Although paclitaxel inhibited MCS formation and reduced cell viability, its cytotoxic effect was significantly lower than that of the other tested compounds. It should be noted that the toxicity effect of the drug depended on the applied breast cancer cell type [89]. An interesting study was presented by Lee et al.; they generated uniform-sized spherical microgels encapsulated with MDA-MB-231, MCF-7, or SKBR-3 breast tumor cells [90]. Significantly, microgel stiffness regulated spheroid growth by imposing growth-induced compressive stresses, which altered cell morphology, cytoskeletal organization, and intracellular tension. Stiffer microgels generated higher mechanical confinement, leading to reduced proliferation, changes in spheroid elastic modulus, and activation of mechanosensitive pathways associated with actin remodeling and ROCK-mediated contractility. At the cellular level, this mechanical interplay modulated mechanotransduction signaling and cell cycle progression, ultimately affecting drug penetration and cellular sensitivity to chemotherapeutics. Consequently, spatially heterogeneous mechanical stresses within spheroids produced distinct cellular mechanical states, contributing to differential therapeutic responses. The obtained spheroids not only indicated that the mechanical properties of microgel strongly influence tumor physiology and spheroid formation depending on the used cell type but also the chemotherapeutic efficacies of paclitaxel and cisplatin. Their cytotoxic effects were heavily influenced by the complex interplay between cell subtype and 3D mechanical microenvironment [90]. The homotypic multicellular tumor spheroid model was developed using microencapsulation in alginate-poly-L-lysine-alginate to test the effect of Mitomycin C, Adriamycin, and 5-fluorouracil [91]. The inhibitory effect of all drugs was approximately two times lower for 3D spheroids than for monolayer MCF-7 cultures [91]. Some drugs can sensitize breast cancer MCS to classic chemotherapeutics, e.g., lapatinib to doxorubicin [87]. Lapatinib, primarily known as a tyrosine kinase inhibitor of two important receptors, EGFR (Epidermal Growth Factor Receptor) and HER2 (Human Epidermal Growth Factor Receptor 2), downregulates the expression of the ABC transporters MDR-1 and BCRP (breast cancer resistance protein), thereby leading to increased intracellular accumulation of doxorubicin. This effect was boosted by increased reactive oxygen species generation in MCF-7 MCS induced by treatment [87]. LQB-223 (11a-N-Tosyl-5-deoxi-pterocarpan) is an example of a drug that can reverse the resistance to doxorubicin of MCF-7 and MDA-MB-231 breast cancer cells spheroids [92]. It significantly decreased cell viability, spheroid size, and cell migration. Moreover, the cytotoxic effect was minimal on the non-neoplastic cells [92]. Pyrrolo[2,3d]pyrimidine-based microtubule-depolymerizing agent (PP-13) that binds to the colchicine site of β-tubulin exhibited anticancer properties in 4T1 breast cancer spheroid cells [93]. It induced a mitotic blockade and apoptosis, reducing cell proliferation, metastatic invasiveness, and the sizes of paclitaxel-refractory orthotopic 4T1 breast tumors, as well as the global metastatic load [93].

#### 3.1.2. Analysis of Cell Sensitivity to Targeted Therapy Drugs on 3D Homotypic Breast Cancer Spheroid Models

Another class of drugs tested on MCS is targeted therapy drugs that attack a specific molecule in cancer cells, usually one that helps them grow. In contrast to conventional chemotherapeutics, which also harm healthy cells, the targeted drug targets cancer cells more precisely, resulting in fewer side effects. Information on their effectiveness obtained during studies using MCS is very valuable.

Apatinib, primarily known as an inhibitor of Vascular Endothelial Growth Factor Receptor 2 (VEGFR2), reduced tumor spheroid formation, as evidenced by a decrease in the size and number of tumor MDA-MB-231 spheroids compared to untreated cells [94]. Additionally, in 2D cell culture, Apatinib induced changes in the migration and invasion of MDA-MB-231 cells, regulated cell morphology and cell cycle, and triggered apoptosis in these cells. Apatinib also decreased the level of p-p65 and p65 proteins within the NF-κB signaling pathway, while increasing the activation of p38, p-p38, JNK, and p-JNK in the MAPK signaling pathway [94]. Some new generation drugs, e.g., Rac inhibitor EHT-1864, PI3K inhibitor AZD6482, maritoclax (an inhibitor of antiapoptotic protein Mcl-1), and erlotinib had a significantly better penetration and inhibition effect on the breast cancer cells spheroids, including MCF-7, T47D, BT474, MDA-MB-231-H2N, and MDA-MB-468, compared to conventional 2D cultures [95]. These breast cancer spheroids grow into a new oxime-crosslinked hyaluronan (HA) hydrogel, maintaining a gene expression profile most similar to that of tumor xenografts (based on an analysis of a pan-cancer gene expression profile comprising 730 genes), in contrast to Matrigel-based spheroids or conventional 2D culture [95]. Moreover, primary human patient tumor luminal B breast cancer cells were significantly more sensitive to maritoclax when cultured in HA–oxime spheroids than those cultured on 2D surfaces, indicating further potential for the HA–oxime-based 3D culture method in personalized medicine [95]. The spheroids formed from primary mammary adenocarcinoma (MMTV-PyMT) were treated with a panel of drugs, including rapamycin, BEZ235, MK2206, and flavopiridol, indicating that effective drug treatment was achieved only with the AKT inhibitor MK2206 and the CDK inhibitor flavopiridol, which reduced the size of the spheroids [96]. Moreover, the applicability of the obtained model was also tested using BT549 and MDA-MB-231 breast cancer cells. Additionally, this study indicated intra-tumor–spheroid heterogeneity, which influences drug efficacy [96]. It was indicated that PI3K p110δ-selective inhibitor (IC87114) and Vps34-selective inhibitor (Vps34-IN1) decreased proliferation, culture growth, invasiveness, and migration of hormone-responsive (MCF-7) and triple-negative (MDA-MB-231) mammary tumor epithelial cells, including a reduction in the growth of spheroids formed by these cells [97].

#### 3.1.3. Analysis of Cell Sensitivity to Drugs Delivered in Nanoparticles on 3D Homotypic Breast Cancer Spheroid Models

The application of nanoparticles (NPs) for drug delivery offers numerous benefits, including protection of the drug from degradation, enhancement of drug solubility and stability, prolongation of circulation, controlled/sustained release, and targeted drug delivery, which potentially reduces side effects and improves overall drug efficacy. Despite the many advantages of using NPs for drug delivery, their application requires an examination of their uptake, penetration, and toxicity. A 3D tumor spheroid model is a valuable tool for such studies.

Yu et al. developed mitochondrial targeting topotecan-loaded liposomes to overcome acquired drug resistance resulting from the drug-induced overexpression of ABC transporters on the cancer cell membrane, as well as intrinsic drug resistance stemming from the apoptotic resistance inherent to the mitochondria of cancer cells [98]. For NP construction, Dequalinium (DQA) was used (a lipophilic cationic compound selectively accumulated into the mitochondria of cancer cells) and D-a-tocopheryl polyethylene glycol 1000 succinate (TPGS1000) to increase chemotherapeutic efficacy by inhibiting the drug efflux of ABC transporters. Due to DQA and TPGS1000 presence in the membrane of liposomes, the mitochondrial targeting and inhibition of drug efflux were increased. The mitochondrial-targeted topotecan-loaded liposomes demonstrated a significant inhibitory effect on both MCF-7 and drug-resistant MCF-7/adr spheroids. Moreover, these targeted liposomes exhibited improved accumulation into MCF-7/adr cell xenografts in mice compared with free topotecan and traditional topotecan-loaded liposomes. Additionally, they were highly effective in treating resistant MCF-7/adr cell xenografts and displayed significant anti-metastatic activity in the naturally resistant B16 melanoma metastatic mouse model [98].

Multiple modifications of drugs or application of drug carriers were proven to improve their ability to penetrate neoplastic cells in spheroids. The in vitro study on 4T1 homotypic spheroids indicated that charge-reversal and reduction-responsive histidine-grafted chitosan-lipoic acid NPs (HCSL-NPs) exhibited excellent penetration at extracellular pH [99]. Folate-targeted polymersomes (vesicles of amphiphilic polymers) programmed to release the encapsulated drugs rapidly at cytosolic concentrations of glutathione were developed to deliver doxorubicin and gemcitabine [100]. These drug carriers were increasingly uptaken by three-dimensional MCF-7 spheroid cultures, indicating a stronger cytotoxic effect of doxorubicin and gemcitabine compared to drug treatment without a carrier [100]. The chitosan oligosaccharide–stearic acid micelles conjugated with doxorubicin can disrupt the tumor microenvironment, reduce systemic side effects, enhance in vitro internalization efficiency, and penetrate the MCF-7 spheroid core, primarily through intercellular channels in peripheral layers, overcoming physical barriers, such as cell–cell and cell–matrix interactions [101]. This allowed for the achievement of a higher intracellular doxorubicin concentration. Thanks to chitosan oligosaccharide-g-stearic acid micelles that accumulate inside the cell, doxorubicin can aid in the efflux by ATP-binding cassette (ABC) transporters, which are highly expressed in MCF-7 cells [101]. pH-releasing liposomal cisplatin penetrated MDA-MB-468 and MDA-MB-231 spheroids significantly better compared with the free cisplatin, which resulted in decreasing spheroid volumes [102]. Curcumin-loaded micellar systems penetrated well inside the MDA-MB-231 spheroids, presenting dose-dependent cellular uptake, cytotoxicity, and significant inhibition of spheroidal growth, diameter, and cell proliferation [103].

### 3.2. Application of Heterotypic 3D Breast Cancer Spheroid for Testing Drug Activity

Heterotypic tumor spheroids are advanced 3D cell cultures that contain cancerous and non-cancerous cells and the ECM [26]. Studies on various cells co-cultured into the form of spheroids provided details about TME, including the impact of ECM communication, hypoxia, pH gradient, nutrient access, and drug permeability [104]. Heterotypic tumor spheroids have significant research value because they more accurately mimic in vivo conditions than homotypic equivalents [26].

Brancato et al. indicated that due to sufficient penetration into nuclei of 3D-cultured cells in homotypic MCF-7 spheroids, doxorubicin was highly effective [105]. However, no inhibitory effect on cell proliferation was observed after treating heterotypic CAF/MCF-7 spheroids with doxorubicin; the drug’s effect was strictly limited to the outer cell layers and did not influence the inner spheroid. The obtained results demonstrated that heterotypic cultures with stromal fibroblasts exhibit significantly higher drug resistance than homotypic cultures in the spheroid model, highlighting the importance of having a multicellular heterotypic model to replicate the cellular interaction [105].

The spheroids from MDA-MB-231 breast cancer cells and fibroblasts, using collagen as the matrix, were successfully developed [27]. The research revealed that both CXCR4 (Chemokine Receptor 4), presented on cancer cells, and CXCL12 (Chemokine Ligand 12), produced by fibroblasts, must be present in the TME for cancer cells to invade the ECM via the activation of the oncogenic MPK pathway. Using an established heterotypic spheroid model, it was demonstrated that blocking tumor–stromal interactions (with a potent CXCR4 antagonist, AMD3100) inhibits cancer cell invasiveness, which represents an interesting potential treatment strategy [27].

An interesting study was presented by Ehsan et al., where a prevascularized tumor (PVT) model was constructed to investigate the early events of solid tumor progression [106]. The primary human endothelial and tumor cells formed a heterotypic spheroid, which was embedded in a fibrin matrix containing fibroblasts. Although the main research was performed on colon cancer SW620 cells, the adaptability of the PVT spheroid model was examined on several cancer cell lines, including the breast cancer MDA-MB-231 cell line. The obtained model enables the study of mechanisms of vessel formation, the process of vascularization within the spheroid, and the intravasation of tumor cells. The research performed on the obtained heterotypic spheroid model indicated that the intravasation of tumor cells was enhanced under low oxygen conditions and was also dependent on the key EMT transcription factor Slug, which represses E-cadherin expression and increases the mobility of cancer cells [106]. The PVT model represents a significant advance in mimicking human tumors in vitro, enabling the testing of drugs targeting angiogenesis, intravasation of tumor cells, and their migration.

Hsieh et al. investigated a nanodrug engineered to respond to multiple biological or environmental triggers using spheroids formed by co-culturing MDA-MB-231 breast cancer cells with dermal fibroblasts [107]. The study demonstrated that doxorubicin’s ability to penetrate the spheroid structure was dependent on the presence of vismodegib. Vismodegib is a drug that binds with the SMO receptor and loosens the dense stroma by down-regulating the Hh signaling pathway, creating a beneficial microenvironment for NPs and doxorubicin penetration. Moreover, in the presence of vismodegib, cell viability was lower than in the control group, confirming the deeper penetration and therapeutic effect of doxorubicin [107]. The presented data indicated that a heterotypic 3D spheroid, mimicking the cancer cell–stroma arrangement, generated a barrier that plays a crucial role in drug response.

Further examples of anticancer drugs tested on breast cancer cell spheroids and a summary are presented in Table 1.

### 3.3. Application of BCSC-Based Spheroids for Testing Drug Activity

The role of BCSCs in the development and progression of breast cancer makes them an important target for anticancer therapy. It is crucial to understand their role in the development of drug resistance during cancer treatment and comprehend their vulnerability, which could become a therapeutic target. The application of the 3D BCSC-based spheroid model could facilitate basic research and the testing of drug activity.

MCSs derived from human mammary epithelial cells are called mammospheres. They are a specialized subtype of spheroids used to study culture and expansion of BCSCs [69,112,113,114]. Cells included in the mammospheres are representative of cancer-initiating cells, as they are able to express stem cell markers and form xenograft tumors [112]. The CD44^high^/CD24^low^ or aldehyde dehydrogenase-positive (ALDH^+^) cells were also able to form mammospheres [68,69,115]. Mammospheres were used to investigate the particular impacts of chemical compounds on BCSCs [116]. Specifically, Sulforaphane, a natural compound derived from broccoli/broccoli sprouts, reduced the size and number of primary mammospheres by 8- to 125-fold and 45% to 75%, respectively [116]. Lamb et al. described that therapeutically targeting protein synthesis in mammospheres (using puromycin, rapamycin, or methionine restriction) is sufficient to inhibit the proliferation and survival of CSCs, indicating a potentially effective strategy for eliminating BCSCS [117].

Natural pharmaceutical products targeting BCSCs, such as triterpene acid, andrographolide, and gomisin M2, were tested in MCSs and were shown to restrain mammosphere growth [118,119,120]. Specifically, andrographolide inhibits the activity of human BCSCs in a dose-dependent manner [118]. Synthetic compounds targeting BCSCs, including doxycycline, a ferutinin analogue, and WX2-43, also reduced the viability of BCSCs in mammospheres [121,122,123]. Additionally, tannic acid and propofol each reduced mammosphere formation in the MCF-7 cell line [124,125].

Antibody-based drugs demonstrate high specificity for tumor cell surface antigens. The monoclonal antibody 602.101, which targets Notch-1, reduces the MDA-MB-231 cancer stem-like subpopulation, induces cell apoptosis, and disrupts mammosphere formation [126]. Additionally, cell therapy enhances immune response and immune competence [127]. TEM8 CAR-T cells, for example, effectively eliminate BCSCs and inhibit the formation of mammospheres in triple-negative breast cancer [128].

The application of nanoparticles was also investigated in the delivery of drugs for BCSC cells. The diblock copolymer nanoparticles loaded with decitabine, co-administered with nanoparticles containing doxorubicin, reduced the proportion of MDA-MB-231 cancer stem cells with high ALDH activity in the mammospheres [129]. This study indicated that decitabine, a DNA hypermethylation inhibitor, is a potent compound for overcoming drug resistance by CSCs.

MCS-based models for studying cancer stem cells have demonstrated significant potential in the development of novel anticancer therapeutic strategies, including immunotherapeutic targeting of CSCs, Notch signaling modulation, and epigenetic interventions [130,131]. Extensive research findings support the importance of CSCs in combating cancer.

## 4. Multicellular Spheroids as a Platform for Personalized Breast Cancer Treatment

As the 21st century advances, so does the field of personalized or precision medicine, dubbed by some as the “medicine’s new frontier” [132]. This branch is based on the idea that diverse genetic variations between individuals, their distinct environments, and their heterogeneous diseases require different treatment strategies. In conclusion, they should be crafted based on individual, patient-specific information (multi-omic analysis in particular) [133,134]. This idea is particularly prominent in the field of oncology, including breast cancer treatment. The majority of drug regimens are based on each patient’s tumor-specific genetic profile [135]. While challenges regarding the development of specific guidelines remain ahead for researchers, fully personalized medicine appears to be inevitable.

**Figure 2:**
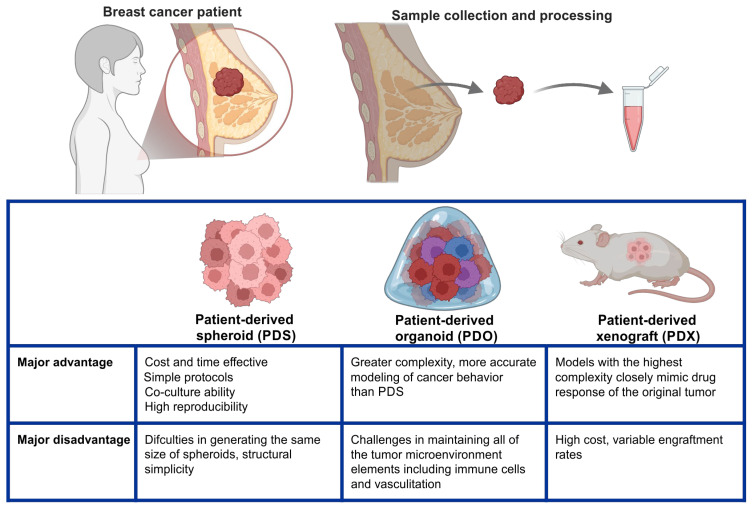
Patient-derived cell culturing techniques in a drug sensitivity profile screening. In principle, these methods require a biopsy of the tumor and administration of cancer tissue into a culture platform. Next, the model is subjected to various treatments. These methods are potentially a valuable tool that can aid physicians in choosing the optimal chemotherapeutics. They may allow for speeding up the therapeutic process and spare the patient the unnecessary discomfort of an ineffective drug regimen. Created in BioRender. KBM, K. (2025) https://BioRender.com/5ij6bo2 (accessed on 18 December 2025).

Drug resistance is one of the most complex and important problems in breast cancer, as the most deadly ones are multidrug resistant (for example, triple negative breast cancer) [136]. To combat this, the researchers developed many prognostic models based on patients’ histories and tumor characteristics [137]. A new form of such models may include patient-derived techniques, including implantation of biopsied cancer tissue into a mouse (patient-derived xenograft, PDX) [138], culture of cancer cells within a scaffold into an organoid form (patient-derived organoids, PDOs) [139], or culture it using 3D spheroid techniques (patient-derived spheroids, PDSs) [140]. Figure 2 summarizes the patient-derived cell treatment techniques useful for screening drug sensitivity profiles. The models are subject to various drug regimens, and ultimately, an effective treatment tailored to the patients’ tumor genetic profile may be established. These techniques may enable the development of more efficient and precise strategies. Multicellular spheroids grown using different techniques may serve as a valuable tool in this area of breast cancer treatment [141]. Hofmann et al. have successfully grown patient-derived breast cancer spheroids with the hanging drop technique. Spheroids were generated from surgically removed breast cancer tissue and co-cultured with normal human fibroblasts in a 96-well plate format. After 4 days of incubation, samples were subjected to commonly used chemotherapeutic drugs (such as paclitaxel, epirubicin, fluorouracil) and to metformin. Spheroids’ response was assessed by measuring ATP levels and spheroids’ size. The spheroids responded with a decrease in size and a drop in ATP levels when subjected to chemotherapeutics. When exposed to metformin, samples exhibited no changes in vitro [142].

While the Hofmann et al. study was focused on developing the method to generate spheroids from human breast cancer tissue (achieving a high success rate at 87%) [140], Halfter et al. conducted a prospective cohort study to evaluate whether patient-derived spheroids can predict responses to neoadjuvant therapy [141]. They obtained biopsied tumor samples from 78 patients eligible for neoadjuvant treatment and grew these samples into spheroids using a modified liquid overlay technique. The spheroids were then cultured with recommended combinations of chemotherapeutics (anthracycline, paclitaxel or docetaxel, cyclophosphamide, carboplatin, trastuzumab), matching the treatment regimen of each patient. Cell survival rates were compared to patients’ actual response to treatment. Patient’s response was defined as pathological complete response (pCR) determined after the completion of chemotherapy and after surgery. A proposed cutoff to predict pCR in patients was established at 35% cell survival. The results demonstrated that the spheroids had a high sensitivity of 95.5% and a specificity of 80.4% for predicting a pathological complete response to neoadjuvant chemotherapy [141].

In another study by Halfter et al., it was demonstrated that drug sensitivity testing on patient-derived spheroids closely aligned with recommended treatment guidelines. In contrast, traditional cell lines did not yield similar results [143]. The spheroids were generated from various types of tissue, including primary tumors, metastases in lymph nodes, and others. After the biopsy, samples were processed mechanically and enzymatically to create a single-cell suspension. Isolated cells were cultured on a 96-well plate to generate spheroids. Treatment efficacy was also measured using an ATP assay to quantify cell survival. An interesting aspect of this study was testing different treatment options on spheroids generated from various metastatic sites (such as lymph node metastases or the peritoneal cavity) and comparing the results with those from spheroids of the primary tumor. Although the results varied widely, the anthracycline–taxane treatment showed the best response. In this study, the response of patient-derived spheroids to tested chemotherapeutics mirrored clinical responses of breast cancer patients to the same regimens. The study also compared patient-derived spheroids with established cell line spheroids (such as MCF7, T-47D, HCC1143, and others). The latter exhibited consistently lower cytostatic responses to chemotherapeutics. Some cell lines (such as HCC1143) were found unsuitable for drug screening models due to their inconsistent results (large scatter of results) [143]. The differences in response may be attributed to the heterogeneity of tissue-generated spheroids and the homogeneity of cell line spheroids. These findings highlight the important role of the tumor microenvironment in mediating a treatment response [143].

Recently, Chen et al. demonstrated that combining patient-derived spheroids with RNA sequencing can quickly and accurately predict patients’ clinical responses to treatment [144]. This approach allows for the assessment of drug sensitivities for each patient within 10 days [144]. They employed proliferation-based gene expression analysis to evaluate tamoxifen sensitivity in an ex vivo setting. The RNA sequencing of samples was performed, evaluating the most common varying transcripts triggered by endoxifen and 4-hydroxytamoxigen (primary metabolites of tamoxifen). Additionally, they found that using a superficial scraping technique to obtain tissue samples significantly increases the number of patients who can be enrolled in the study, as it enables the use of this method on small tumors, even those as small as 3 mm [144].

There are currently not many reports on the use of multicellular spheroids as a platform for personalized breast cancer treatment, but studies that extend beyond breast cancer can be indicated. In pediatrics, a two-year pilot study called INFORM was conducted, in which Peterziel et al. examined the effectiveness of ex vivo drug sensitivity profiling using patient-derived spheroids created from various tumor types, including primary brain neoplasms. A drug screening protocol with a quick turnaround time of under three weeks was established. The INFORM pipeline demonstrated at least one drug hit in 72% of samples and could not find any sensitive drugs in 28% of samples. The established protocol accurately identified drug candidates in approximately 81% of samples where RNA sequencing and whole-exome sequencing (WES) could not pinpoint a suitable therapeutic target. This finding suggests that ex vivo drug sensitivity analysis based on patient-derived spheroids might add clinically relevant information for clinicians responsible for choosing the best drug regimen [145]. In ovarian cancer, Shuford et al. developed a model capable of predicting patients’ clinical response to drug regimens within seven days for those newly diagnosed with ovarian cancer undergoing primary debulking surgery. The model demonstrated a sensitivity of 87% and a specificity of 100%, and it performed similarly in a neoadjuvant treatment group [146]. The superiority of patient-derived spheroids over cell line spheroids in terms of drug screening efficacy highlights the importance of the tumor microenvironment and its influence on the tumor’s response to treatment.

It is worth pointing out that not only cancer cells derived from the patients’ solid tumors can be used for establishing a 3D tumor model and constitute a target for therapy. Patient-derived circulating tumor cells (CTCs) or CAFs were also examined in breast cancer spheroids, indicating a promising platform for personalized therapy design [109,110].

### 4.1. The Limitations of the Use of Spheroids in Individualized Anticancer Therapies

Numerous examples of the therapeutic use of spheroids have been reported to date. Although cancer spheroids have the potential to be valuable tools in cancer treatment in the future, they currently face significant challenges. Many protocols for spheroid formation require time to form stable, compact spheroids before they can be used for drug resistance assays. In contrast to 2D cultures, where cells can be ready for testing in just one day, spheroids often require several days of pre-culture before drug exposure [147].

The biological conditions of a real organism, including vascularization, the role of immune cells in the tumor microenvironment, and the accurate delivery of nutrients, oxygen, and therapeutic agents to tumor cells, are challenging to recreate [148]. These factors, combined with the specific structure of spheroid culture itself, may heavily influence drug penetration during testing. As a result, this could lead to misleading conclusions regarding the efficacy of standard therapies based on patient-derived spheroid testing [148].

Currently, although spheroid cultures remain useful and may play a significant role in individualized medicine in the future, only therapies that have passed animal testing are officially approved for anticancer treatment [149]. Despite their potential, patient-derived spheroids have not yet been implemented in clinical settings. The lack of rigorous and standardized protocols results in poor reproducibility of experiments, making it difficult to compare results of well-conducted studies. Below, we list the most crucial limitations, particularly regarding method standardization.

#### 4.1.1. Batch-to-Batch Inconsistency

The term refers to variability in spheroid size, structure, and functional behavior that occurs between independently prepared batches, even when identical protocols are followed. This variability results from minor differences in cell state, seeding conditions, the ECM composition, plate characteristics, or the expertise and precision of the scientists involved [18,150]. Currently, batch-to-batch inconsistency remains a significant limitation to reproducibility in multicellular spheroid research [151].

#### 4.1.2. Sensitivity to ECM Composition

With numerous chemical components, the specific stiffness and unique influence on cell functioning and proliferation, as well as the effectiveness of drug transmission, the ECM plays a fundamental role in spheroid formation studies. Accurately recreating the ECM composition appropriate for specific tumor cell types remains a substantial challenge in this field [18]. As mentioned above, a study by Lee et al. indicated that the cytotoxic effect of the drug was significantly dependent on the interaction between the mechanical properties of the microgel and the breast cancer cell subtype [90]. Additionally, the depot of molecules embedded into the ECM is of special importance, as it has been shown that cancer patient outcomes can be predicted from the ECM composition. For example, tumors with high expression of protease inhibitors correlate with a positive outcome. In contrast, tumors with a high expression of integrins and MMPs are associated with poor prognosis and increased risk of recurrence [24]. The awareness of the importance of EMC represents an important step forward in personalized medicine. For instance, a cell-free patient-derived scaffold can effectively replicate the native TME [21]. The recellularized patient-derived scaffold has been shown to exhibit increased resistance to standard chemotherapeutic agents compared to cancer cells grown in 2D conditions [21].

#### 4.1.3. Differences in Necrotic Core Formation

In a 3D spheroid, as the diameter increases, the diffusion of oxygen and nutrients to the inner parts of the cells becomes limited. The nutrient deprivation and hypoxia in the most central region of the spheroid lead to cell necrosis. The size and properties of the necrotic core vary, influencing the outcomes of spheroid studies [152].

#### 4.1.4. Seeding Density and Methodology

Seeding density refers to the initial number of cells introduced into each well or culture microenvironment to initiate the formation of spheroids. Studies show that the seeding density clearly determines the size and, therefore, the properties of the formed spheroids, which can lead to significant differences in the results of similar experiments [153]. Therefore, it is a critical parameter that affects the scientific value of experiments on multicellular spheroids. Moreover, the specific methodology selected for the study, such as forced floating, suspension culture, hanging drop, etc., also plays an important role in the outcome [153].

#### 4.1.5. Plate Geometry

The geometry of the plate significantly affects how cells settle, aggregate, and compact, which in turn influences a spheroid’s form, the speed of its formation, and the shape and size it ultimately reaches. Research has shown that using U-shaped vessels with a round bottom led to better formation of a single and compact spheroid culture [150,154]. In contrast, flat-bottomed wells tend to result in multiple separate spheroid cultures forming, which vary in size [154]. Additionally, it has been observed that the depth of the well is important; as the depth increases, the size of the spheroids tends to decrease [155,156].

## 5. Personalized Breast Cancer Treatment—Comparing PDSs, PDOs, and PDXs

PDXs (patient-derived xenografts), PDOs (patient-derived organoids), and PDSs (patient-derived spheroids) as models exhibit and resemble cancer in vivo to different degrees. Each of those models has its own advantages and disadvantages. Based on current knowledge, it is suggested that each method can be beneficial, but for slightly different purposes. For example, PDXs find their application in comprehensive drug evaluation, PDOs in high-throughput drug screening, and PDSs in large-scale initial drug screening [157,158,159,160,161,162,163,164,165]. The detailed descriptions of PDX, PDO, and PDS models can be found in recent reviews [157,158,159,160,161,162,163,164,165], and a brief comparison of PDXs PDO, and PDS models is presented in Table 2.

Spheroids differ from 2D cultures by capturing proper 3D organization, spatial gradients, and more realistic cell signaling. Compared to organoids, they are simpler and more controllable, allowing more precise analysis of specific mechanisms while remaining more physiologically relevant than flat cultures. Unlike animal models, spheroids are human-cell-based, scalable, and experimentally tractable, uniquely revealing how 3D structure and collective cell behavior influence biology and treatment response. Unfortunately, due to still-limited data from spheroid-based studies and significant disparities between studies using classical cytotoxic drugs and newer therapies, such as monoclonal antibodies or antibody–drug conjugates, it is not possible to determine which class of drugs can be more effectively evaluated using spheroids. For the same reasons, there is a significant question as to whether drug sensitivity results obtained from spheroids can be reliably translated into clinical therapy in humans. Nevertheless, there is hope that applying this form of personalized diagnostics for assessing drug sensitivity in cancer patients, including breast cancer patients, may, in the future, lead to the expected therapeutic benefits.

## 6. Conclusions

Drug resistance is a significant cause of treatment failure, not only in breast cancer. The development of a simple, reproducible, time-efficient, and reliable method for drug screening is highly desirable. Multicellular spheroids offer certain advantages; they do not require a scaffold or host, reducing costs, time, and ethical dilemmas. However, they have specific drawbacks, the main being structural simplicity and the difficulty in replicating spheroids of the same size and cell number. A solution is to develop culture schemes that allow for the replication of more similar spheroids.

Furthermore, interactions within the TME directly influence resistance to chemotherapeutic drugs. Additional factors include tumor impermeability due to physical barriers and immune reactions occurring within the tumor. Understanding the TME, particularly its key components (such as CAFs, TAMs, and BCSCs), is crucial for comprehending tumor behavior and accurately selecting therapies through drug screening. Therefore, developing heterotypic tumor spheroids to study this issue in detail may be the most effective approach.

Furthermore, combating chemoresistance is challenging due to the complexity and heterogeneity of tumors in individual patients. Personalized oncology presents a promising approach for enhancing cancer treatment. Patient-derived multicellular spheroids, as a model for testing various chemotherapeutic agents, can enable physicians to identify and implement appropriate treatment regimens. Based on initial evidence, PDSs can be a valuable tool in drug screening [141,142,143]. However, standardization of the method and the development of models that better reflect in vivo interactions between tumor cells and other cell types, i.e., the development of a heterotypic spheroid, are necessary. Developing strategies that enable treatment selection tailored to the genetic and phenotypic profile of a patient’s tumor while simultaneously assessing drug sensitivity is crucial not only in breast cancer treatment.

## Figures and Tables

**Table 1:** Examples of anticancer drugs tested on breast cancer cell line-based spheroids.

Molecule Drug	Function of The Drug	Breast Cancer Cell Line	Type of Spheroid	Method of Cell Culture	Results	Ref.
IC87114 Vps34-IN1	PI3K p110δ inhibitor Vps34 inhibitor	MCF-7 MDA-MB-231	Homo	Breast cancer cells were mixed with VitroGel-3D-RGD matrix and seeded into a 24-well plate. After solidification, the spheroids were cultivated into spheroids plating medium for up to 15 days.	In spheroid models, the inhibitors significantly reduced spheroid growth, with greater efficacy observed in the MCF-7-spheroids than in the MDA-MB-231-spheroids. Findings compared and confirmed that 3D models are preferable to 2D cultures in drug testing.	[97]
Doxorubicin (DXR) and cisplatin (CDDP) in lipid nanoparticles (NPs)	Cytotoxic DXR and CDDP loaded into pH-responsive NPs	MDA-MB-231 DXR-resistant MDA-MB-231 (DXR-Res-231)	Homo	Breast cancer cells were mixed with Matrigel and seeded onto poly-HEMA-coated U-bottom 96-well plates. Spheroids were cultivated up to 9 days.	Spheroids developed interstitial pH profiles that ranged from 6.5 (center) to 7.4 (periphery). Two pH-responsive NPs were applied for drug release and attachment to the negatively charged tumor ECM in the acidic TME. Environmentally responsive nanoparticles enhanced the bioavailability of DXR and CDDP at tumor sites. The combined treatment with DXR-NPs and CDDP-NPs exhibited greater inhibition of outgrowth spheroids compared with treatment with each drug alone, particularly in spheroids formed by DXR-Res-231 cells.	[108]
Mitomycin Adriamycin 5-fluorouracil	Cytotoxic	MCF-7	Homo	Breast cancer cells were enclosed in alginate-poly-L-lysine-alginate (APA) microcapsules to form microencapsulated multicellulartumor spheroid (MMTS). Spheroids were cultivated for up to 5 days.	Drugs caused a reduction in the size of cell spheroids and a decrease in cell viability. The inhibition of cell viability in MMTS was much lower than that in monolayer cell culture.	[91]
LQB-223	Inductor of apoptosis, inhibitor of cell proliferation and migration	MCF-7 MDA-MB-231	Homo	Breast cancer cells were seeded onto flat-bottom 96-well plates coated with agarose to form spheroids. Cell spheroids were cultivated until they reached a diameter between 300 and 500 µm and then were used for experiments.	In cancer spheroids, LQB-223 decreased cell viability, reduced tumor volume, and impaired cell migration.	[92]
Pertuzumab Trastuzumab Lapatinib Gemcitabine Tamoxifen	Cytotoxic Her-2 inhibitor TKI SERM	Circulating tumor cells isolated from newly diagnosed breast cancer patients	Homo	Circulating tumor cells (CTCs) and CTC-WBC clusters were isolated from a breast cancer patient’s blood using the LIPO-SLB platform. Isolated CTCs were cultured ex vivo to form spheroids for 6 days.	Common chemotherapeutics (anthracycline-, taxane-, and platinum-based, alone or in combination) were evaluated for their impact on CTC–spheroid viability. Drug sensitivity testing revealed effective treatment options for nine of thirteen patients.	[109]
Screen of the Approved Oncology Drug Collection (89 drugs)	EGF receptor inhibitors, broad-spectrum kinase inhibitors	BT-474	Homo and Hetero	BT-474, epithelial cell line (MCF10A), human fibroblasts HF (Hs.58), and human umbilical vein endothelial cells HE (HUVEC) were cultured as monolayers and spheroids. Cells formed spheroids spontaneously in 96-well ultralow attachment plates and were cultivated for 5 days. For 3D hetero co-cultures, BT-474:HF:HE cells were mixed at a ratio of 2:1:1, respectively.	Only under 3D culture conditions were homo BT-474-based spheroids more sensitive to lapatinib, gefitinib, or dasatinib than control epithelial cell (MCF10A)-based spheroids, in contrast to 2D cell culture conditions. Twelve drugs (lapatinib, gefitinib, dasatinib, ixabepilone, paclitaxel, vinorelbine, rapamycin, azacitidine, vinblastine, vincristine, taxotere, nilotinib) exhibited greater selectivity for the 3D BT-474 + HF + HE spheroids than the control 3D HF + HE spheroids. The 3D hetero spheroids were identified as valuable tools for identifying clinically useful drugs.	[32]
Cisplatin	Cytotoxic	BT474 T47D MDA-MB-231 SK-BR-3	Hetero and homo	The MCTSs contain tumor cells, CAFs, macrophages, and endothelial cells Ea.hy926. CAFs were isolated from breast cancer biopsy specimens. Macrophages were differentiated from the monocyte cell line THP-1. Cells were seeded onto an ultralow attachment 96-well plate and cultured for up to 5–7 days to form multicellular tumor spheroids.	MCTSs displayed different special arrangements of CAFs, endothelial cells, and macrophages, depending on the cancer cell line. Cancer cell lines showed different invasive potential in MCTS-BT474 and MDA-MB-231, showing the highest potential, and T47D and SK-BR-3, showing the lowest. MCTSs induced a higher degree of macrophage polarization than the co-culture of cancer cells with macrophages. For BT474, T47D, and SK-BR-3, the 3D MCTSs were more resistant to cisplatin than the corresponding 2D tetraculture models.	[110]
AMD3100	Potent antagonist of CXCR4	MDA-MB-231	Hetero	CXCR4^+^ TNBC cells formed spheroids in dextran onto the 384-well ultralow attachment plate covered with PEG. After 24 h, cancer spheroids were covered with collagen containing fibroblasts (HMFs or CXCL12 + HMFs) and incubated for up to 5 days.	After 24 h of incubation with normal fibroblasts, the invasion of cancer cells into the ECM was significantly reduced, while incubation with CXCL12-producing fibroblasts promoted cancer invasion. Blocking CXCR4 signaling with ADM3100 maintained the cancer cells as a minimally invasive spheroid. Molecular analysis showed a decrease in p-ERK1/2 levels, confirming the role of the MAPK pathway in TNBC cell invasiveness and the potential of inhibiting tumor–stromal signaling as a therapeutic strategy.	[27]
Axitinib (AXI) Deshydroxy LY-411575 LDC1267 RS-504393 Pomalidomide Pirfenidone ZD-7155 UK 383367 Minoxidil βAPN	TME-targeted antifibrotic and antiangiogenic drugs, including inhibitors of VEGF-R1, -R2, -R3 g-Secretase pan-TAMRTK, CCR2, BMP1, PLOD2, Lysyl oxidase, and TNFa antagonist	MCF7 MDA-MB-435s MDA-MB-231 SK-BR3 ZR75-1 MDA-MB-468	Hetero	Tumor cells with normal human dermal fibroblasts (HDFs) were seeded onto a 96-well plate coated with 1.0% agarose to form multicellular tumor spheroids (MCTSs). To obtain vascularized tumor spheroids (VTSs), tumor cells, HDFs, human umbilical vein endothelial cells (ECs), and monocytic cell THP-1 were seeded onto agarose-coated 96-well plates. Spheroids reached ~300–500 µm and developed a pseudovascular network after 9 days.	Different tumor cell lines generated their own VTS composition and architecture, which were changed after treatment with TME-targeted drugs. After anti-angiogenic treatment (using AXI, LDC1267, DesOH LY-411575), the overall cell viability was not affected; however, the size of VTS was reduced (for AXI/MDA-MB-435s VTSs and DesOH LY-411575/MDA-MB-231 VTSs), and the VTS composition was modified (reduced number of fibroblasts but increased number of tumor cells). AXI strongly decreased the number of CD31 + EC cells and disrupted pseudovascular (PV) networks; LDC1267 and DesOH LY-411575 also reduced PV complexity, with DesOH LY-411575 increasing PV volume in MDA-MB-231 VTSs. After treatment, tumor cells were localized closer to PV. ECM-targeting drugs (βAPN, minoxidil, pirfenidone, pomalidomide, RS-504393, ZD-7155, UK 383367) also modulated VTS architecture. MDA-MB-231 and MDA-MB-435s VTSs showed significant shifts in cellular composition, while MCF7 VTSs remained largely resistant. The MDA-MB-435s VTSs and MDA-MB-231-based VTSs were more resistant to paclitaxel (PTX) and cisplatin (CDDP) treatment, respectively, compared to 2D corresponding cultures.	[111]

**Table 2:** A comparison of PDXs, PDOs, and PDSs for drug sensitivity testing for individual patients [144,157,158,159,160,161,162,163,164,165].

	PDXs	PDOs	PDSs
Miscellaneous advantages and disadvantages	- model in vivo - samples of cancer tissue are implanted into an animal model - results of preclinical studies conducted on PDXs can be useful for establishing toxicity and dosing windows; however, they cannot be used to derive pharmacokinetic parameters due to inconsistencies between mouse and human pharmacokinetics - results of preclinical studies conducted on PDXs can accurately predict a patient’s response to treatment - creating a PDX model involves animal testing and various sample processing techniques, making it a complex, expensive, and ethically questionable process - variable success rate of engraftment - creating a PDX model is time-consuming, which is a significant issue because time is often limited for oncological patients	- model in vitro - samples of cancer tissue from surgical resections or biopsies are being used to establish a model, eliminating the need for animal involvement in this process. - generating an organoid only requires a small part of cancer tissue, which does not interfere with oncological treatment - generating a sufficient number of organoids for drug testing is time-consuming - relatively low reliability and reproducibility due to a lack of standardized culture protocols, but when models are established, they are considered reproducible - in terms of labor intensity, establishing a PDO model is less demanding than a PDX and more than a PDS model	- model in vitro - samples of cancer cells grown in 3D spheroids formed primarily via cell-to-cell adhesion (i.e., cell aggregates) - low complexity of the model concerns its reliability - models can be maintained for several weeks, allowing for long-term studies. - the lack of standardized culture protocols is a significant obstacle to this method
Cost, timeline, and scalability	- high cost, more resource-intensive - slow, several weeks to months, depending on the number of passages - poor model efficiency compared to PDO hinders larger-scale studies	- relatively high, but less costly than the PDX model - fast, time-efficient, faster than the PDX model - scalable	- low cost, cost-effective - the fastest, quickest turnaround time - scalable, but standardization is a significant issue that poses as an obstacle to conducting larger-scale studies
Similarity to tumors in vivo	- the most complex model - PDX model demonstrates a high histological resemblance to the original tumor - PDX models provide the most complete TME, including interactions with stroma and other molecules of the body’s system - human cancer cells cultured in mice adapt to the microenvironment, leading to genomic and biomarker alterations that can change their response to drugs - improper reflection of interaction with the immune system, but humanized mice and mice with a reconstituted human immune system are available	- exhibit a high level of similarity to tumors in vivo - mimic the cellular composition, structure, and function of the original tumor - assemble on a scaffold mimicking the ECM, but frequently, the ECM is artificially defined - preserve certain aspects of tumor heterogeneity - PDO models only exhibit tissue fragments and a lack of complete cancer complexity, such as interactions with immune cells, tumor microenvironment, and blood vessels - since tissue samples originate from different patients, the established models can exhibit patient diversity to some extent - similarity to tumors in vivo is greater than in PDS models	- histologically resemble tumors at some points, but less than organoids - can mimic tumor metabolism and present conditions such as hypoxia, along with gradients of oxygen and carbon dioxide - can exhibit subpopulations of cancer stem cells and a tumor-like layered structure, including a necrotic zone - scaffold-free models facilitate cell-to-cell interactions, encouraging cellular alterations, similar to tumors in vivo - show some similarities in protein expression and gene expression profiles, such as in vivo - can simulate interactions between cells and the extracellular matrix - offer a more relevant environment than 2D cell culture models but represent the simplest 3D architecture compared with PDO and PDX models
Feasibility of drug screening for real patients	- presently, PDXs are widely applied in fundamental research and drug development in many institutions and companies - to establish the method, it requires a lot of labor, skills, time, and costs, which constitute a significant obstacle in larger-scale experiments - engraftment rate is unsatisfactory; mostly, the highly malignant tumors are successfully implanted (not all cancer patients can potentially benefit) - the intrinsic cancer property can be altered after a few generations, limiting long-term study. - currently, research is being conducted on drug efficacy in cancer, utilizing both PDXs and organoids derived from PDXs (PDXO model), decreasing overall cost and increasing the high-throughput of the method	- the well-established PDOs are currently considered the most reproducible and robust for drug screening - PDOs can be established from tumors in all grades (all cancer patients can potentially benefit) - can be cryopreserved and recovered - their propagation time is relatively fast - PDOs are less demanding on labor and costs than PDXs, but high skills and experience are required - the responses to anticancer treatment represent only part of a tissue and do not fully reflect the responses of an entire tumor - potentially can be generated for each cancer patient, which can aid in making clinical decisions; however, the time, cost, and technical difficulty of this method still require improvements for its straightforward application	- can mimic the 3D architecture of a tumor and clinically relevant resistance to anticancer treatment - can present responses of cancer cells to anticancer treatments, allowing for tracking of the processes and changes occurring in cells, which are essential for further clinical trials of anticancer drugs - can be cryopreserved and recovered - the lowest cost, time, labor, and technical difficulties compared with PDOs and PDXs, making it easier for implementation in standard laboratory use - as PDSs do not reflect a complete cellular composition and interactions in tumors, limiting the possibility of in-depth studies

## Data Availability

No new data were created or analyzed in this study. Data sharing is not applicable to this article.

## Abbreviations

The following abbreviations are used in this manuscript:

2D	Two-dimensional
3D	Three-dimensional
5-FU	5-fluorouracile
ABC	ATP-binding cassette
AKT	Protein kinase B
ALDH	Aldehyde dehydrogenase
APA	Alginate-poly-L-lysine-alginate
AXI	Axitinib
BCRP	Breast cancer resistance protein
BCSCs	Breast cancer stem cells
CAFs	Cancer-associated fibroblasts
CDK	Cyclin-dependent kinase
CSCs	Cancer stem cells
CTCs	Circulating tumor cells
CXCR4	Chemokine receptor 4
CXCL12	Chemokine ligand 12
ECM	Extracellular matrix
EGF	Epidermal growth factor
EMT	Epithelial–mesenchymal transition
ERα	Estrogen Receptor Alpha
HDFs	Human dermal fibroblasts
HFs	Human fibroblasts
HUVECs	Human umbilical vein endothelial cells
IC87114	PI3K p110δ-selective inhibitor
JNK	c-Jun N-terminal kinase
LQB-223	11a-N-Tosyl-5-deoxi-pterocarpan
MAPK	Mitogen-activated protein kinase
MCS	Multicellular spheroid
MCTS	Multicellular tumor spheroid
MDR-1	Multidrug resistance protein 1
MMTS	Microencapsulated multicellulartumor spheroid
NETs	Neutrophil extracellular traps
NF-κB	Nuclear factor kappa B
NK cells	Natural killer cells
NPs	Nanoparticles
PDOs	Patient-derived organoids
PDSs	Patient-derived sheroids
PDXs	Patient-derived xenografts
PEG	Polyethylene glycol
PI3K	Phosphoinositide 3-kinase
Poly-HEMA	poly(2-hydroxylethylmethacrylate)
PP-13	Pyrrolo[2,3d]pyrimidine-based microtubule- depolymerizing agent
PV	Pseudovascular
TAMs	Tumor-associated macrophages
TIME	Tumor immune microenvironment
TME	Tumor microenvironment
TNBC	Triple-negative breast cancer
RPM	Random positioning machine
Vps34-IN1	Vps34-selective inhibitor
VTSs	Vascularized tumor spheroids

## References

[1] Breast Cancer Statistics and Resources. Breast Cancer Research Foundation. https://www.bcrf.org/breast-cancer-statistics-and-resources/.

[2] Khan S.U., Fatima K., Aisha S., Malik F. (2024). Unveiling the mechanisms and challenges of cancer drug resistance. Cell Commun. Signal..

[3] Thomas R.M., Van Dyke T., Merlino G., Day C.P. (2016). Concepts in Cancer Modeling: A Brief History. Cancer Res..

[4] Robinson N.B., Krieger K., Khan F.M., Huffman W., Chang M., Naik A., Yongle R., Hameed I., Krieger K., Girardi L.N. (2019). The current state of animal models in research: A review. Int. J. Surg..

[5] Akhtar A. (2015). The flaws and human harms of animal experimentation. Camb. Q. Healthc. Ethics.

[6] Breslin S., O’Driscoll L. (2016). The relevance of using 3D cell cultures, in addition to 2D monolayer cultures, when evaluating breast cancer drug sensitivity and resistance. Oncotarget.

[7] Han K., Pierce S.E., Li A., Spees K., Anderson G.R., Seoane J.A., Lo Y.H., Dubreuil M., Olivas M., Kamber R.A. (2020). CRISPR screens in cancer spheroids identify 3D growth-specific vulnerabilities. Nature.

[8] Santini M.T., Rainaldi G., Indovina P.L. (1999). Multicellular tumour spheroids in radiation biology. Int. J. Radiat. Biol..

[9] Weiswald L.-B., Bellet D., Dangles-Marie V. (2015). Spherical cancer models in tumor biology. Neoplasia.

[10] Rimann M., Graf-Hausner U. (2012). Synthetic 3D multicellular systems for drug development. Curr. Opin. Biotechnol..

[11] Vasan N., Baselga J., Hyman D.M. (2019). A view on drug resistance in cancer. Nature.

[12] Gong X., Lin C., Cheng J., Su J., Zhao H., Liu T., Wen X., Zhao P. (2015). Generation of Multicellular Tumor Spheroids with Microwell-Based Agarose Scaffolds for Drug Testing. PLoS ONE.

[13] Horning J.L., Sahoo S.K., Vijayaraghavalu S., Dimitrijevic S., Vasir J.K., Jain T.K., Panda A.K., Labhasetwar V. (2008). 3-D tumor model for in vitro evaluation of anticancer drugs. Mol. Pharm..

[14] Aggarwal V., Miranda O., Johnston P.A., Sant S. (2020). Three dimensional engineered models to study hypoxia biology in breast cancer. Cancer Lett..

[15] Imamura Y., Mukohara T., Shimono Y., Funakoshi Y., Chayahara N., Toyoda M., Kiyota N., Takao S., Kono S., Nakatsura T. (2015). Comparison of 2D- and 3D-culture models as drug-testing platforms in breast cancer. Oncol. Rep..

[16] Bahcecioglu G., Basara G., Ellis B.W., Ren X., Zorlutuna P. (2020). Breast cancer models: Engineering the tumor microenvironment. Acta Biomater..

[17] Rohwer N., Cramer T. (2011). Hypoxia-mediated drug resistance: Novel insights on the functional interaction of HIFs and cell death pathways. Drug Resist. Updat..

[18] Langhans S.A. (2018). Three-Dimensional in Vitro Cell Culture Models in Drug Discovery and Drug Repositioning. Front. Pharmacol..

[19] Jensen C., Teng Y. (2020). Is It Time to Start Transitioning from 2D to 3D Cell Culture?. Front. Mol. Biosci..

[20] Giusti I., Poppa G., D’Ascenzo S., Esposito L., Vitale A.R., Calvisi G., Dolo V. (2022). Cancer Three-Dimensional Spheroids Mimic In Vivo Tumor Features, Displaying “Inner” Extracellular Vesicles and Vasculogenic Mimicry. Int. J. Mol. Sci..

[21] Salemme V., Centonze G., Avalle L., Natalini D., Piccolantonio A., Arina P., Morellato A., Ala U., Taverna D., Turco E. (2023). The role of tumor microenvironment in drug resistance: Emerging technologies to unravel breast cancer heterogeneity. Front. Oncol..

[22] Egeblad M., Nakasone E.S., Werb Z. (2010). Tumors as organs: Complex tissues that interface with the entire organism. Dev. Cell.

[23] Radisky D., Hagios C., Bissell M.J. (2001). Tumors are unique organs defined by abnormal signaling and context. Semin. Cancer Biol..

[24] Quail D.F., Joyce J.A. (2013). Microenvironmental regulation of tumor progression and metastasis. Nat. Med..

[25] Bissell M.J., Radisky D. (2001). Putting tumours in context. Nat. Rev. Cancer.

[26] Fiorini E., Veghini L., Corbo V. (2020). Modeling Cell Communication in Cancer with Organoids: Making the Complex Simple. Front. Cell Dev. Biol..

[27] Singh S., Ray L.A., Thakuri P.S., Tran S., Konopka M.C., Luker G.D., Tavana H. (2020). Organotypic Breast Tumor Model Elucidates Dynamic Remodeling of Tumor Microenvironment. Biomaterials.

[28] Weydert Z., Lal-Nag M., Mathews-Greiner L., Thiel C., Cordes H., Küpfer L., Guye P., Kelm J.M., Ferrer M. (2020). A 3D Heterotypic Multicellular Tumor Spheroid Assay Platform to Discriminate Drug Effects on Stroma versus Cancer Cells. SLAS Discov..

[29] Giraldo N.A., Sanchez-Salas R., Peske J.D., Vano Y., Becht E., Petitprez F., Validire P., Ingels A., Cathelineau X., Fridman W.H. (2019). The clinical role of the TME in solid cancer. Br. J. Cancer.

[30] Li H., Fan X., Houghton J. (2007). Tumor microenvironment: The role of the tumor stroma in cancer. J. Cell. Biochem..

[31] Vakhshiteh F., Bagheri Z., Soleimani M., Ahvaraki A., Pournemat P., Alavi S.E., Madjd Z. (2023). Heterotypic tumor spheroids: A platform for nanomedicine evaluation. J. Nanobiotechnol..

[32] Howes A.L., Richardson R.D., Finlay D., Vuori K. (2014). 3-Dimensional Culture Systems for Anti-Cancer Compound Profiling and High-Throughput Screening Reveal Increases in EGFR Inhibitor-Mediated Cytotoxicity Compared to Monolayer Culture Systems. PLoS ONE.

[33] Lorico A., Rappa G. (2011). Phenotypic Heterogeneity of Breast Cancer Stem Cells. J. Oncol..

[34] Zhang L., Chen W., Liu S., Chen C. (2023). Targeting Breast Cancer Stem Cells. Int. J. Biol. Sci..

[35] Song K., Farzaneh M. (2021). Signaling pathways governing breast cancer stem cells behavior. Stem Cell Res. Ther..

[36] Kim H., Lin Q., Glazer P.M., Yun Z. (2018). The hypoxic tumor microenvironment in vivo selects the cancer stem cell fate of breast cancer cells. Breast Cancer Res..

[37] Singh D., Dhiman V.K., Pandey M., Dhiman V.K., Sharma A., Pandey H., Verma S.K., Pandey R. (2024). Personalized medicine: An alternative for cancer treatment. Cancer Treat. Res. Commun..

[38] Menon G., Alkabban F.M., Ferguson T. (2025). Breast Cancer. StatPearls.

[39] Idrisova K.F., Simon H.U., Gomzikova M.O. (2022). Role of Patient-Derived Models of Cancer in Translational Oncology. Cancers.

[40] Mayer S., Milo T., Isaacson A., Halperin C., Miyara S., Stein Y., Lior C., Pevsner-Fischer M., Tzahor E., Mayo A. (2023). The tumor microenvironment shows a hierarchy of cell-cell interactions dominated by fibroblasts. Nat. Commun..

[41] Mizutani Y., Kobayashi H., Iida T., Asai N., Masamune A., Hara A., Esaki N., Ushida K., Mii S., Shiraki Y. (2019). Meflin-Positive Cancer-Associated Fibroblasts Inhibit Pancreatic Carcinogenesis. Cancer Res..

[42] Fiori M.E., Di Franco S., Villanova L., Bianca P., Stassi G., De Maria R. (2019). Cancer-associated fibroblasts as abettors of tumor progression at the crossroads of EMT and therapy resistance. Mol. Cancer.

[43] Hinshaw D.C., Shevde L.A. (2019). The Tumor Microenvironment Innately Modulates Cancer Progression. Cancer Res..

[44] Joshi R.S., Kanugula S.S., Sudhir S., Pereira M.P., Jain S., Aghi M.K. (2021). The Role of Cancer-Associated Fibroblasts in Tumor Progression. Cancers.

[45] Wu F., Yang J., Liu J., Wang Y., Mu J., Zeng Q., Deng S., Zhou H. (2021). Signaling pathways in cancer-associated fibroblasts and targeted therapy for cancer. Signal Transduct. Target. Ther..

[46] Pontiggia O., Sampayo R., Raffo D., Motter A., Xu R., Bissell M.J., de Kier Joffé E.B., Simian M. (2012). The tumor microenvironment modulates tamoxifen resistance in breast cancer: A role for soluble stromal factors and fibronectin through β1 integrin. Breast Cancer Res. Treat..

[47] Shekhar M.P., Santner S., Carolin K.A., Tait L. (2007). Direct involvement of breast tumor fibroblasts in the modulation of tamoxifen sensitivity. Am. J. Pathol..

[48] An Y., Liu F., Chen Y., Yang Q. (2020). Crosstalk between cancer-associated fibroblasts and immune cells in cancer. J. Cell. Mol. Med..

[49] Barrett R., Puré E. (2020). Cancer-associated fibroblasts: Key determinants of tumor immunity and immunotherapy. Curr. Opin. Immunol..

[50] Barrett R.L., Puré E. (2020). Cancer-associated fibroblasts and their influence on tumor immunity and immunotherapy. Elife.

[51] Ene-Obong A., Clear A.J., Watt J., Wang J., Fatah R., Riches J.C., Marshall J.F., Chin-Aleong J., Chelala C., Gribben J.G. (2013). Activated pancreatic stellate cells sequester CD8^+^ T cells to reduce their infiltration of the juxtatumoral compartment of pancreatic ductal adenocarcinoma. Gastroenterology.

[52] Tan B., Shi X., Zhang J., Qin J., Zhang N., Ren H., Qian M., Siwko S., Carmon K., Liu Q. (2018). Inhibition of Rspo-Lgr4 Facilitates Checkpoint Blockade Therapy by Switching Macrophage Polafrization. Cancer Res..

[53] Ksiazkiewicz M., Gottfried E., Kreutz M., Mack M., Hofstaedter F., Kunz-Schughart L.A. (2010). Importance of CCL2-CCR2A/2B signaling for monocyte migration into spheroids of breast cancer-derived fibroblasts. Immunobiology.

[54] Cohen N., Shani O., Raz Y., Sharon Y., Hoffman D., Abramovitz L., Erez N. (2017). Fibroblasts drive an immunosuppressive and growth-promoting microenvironment in breast cancer via secretion of Chitinase 3-like 1. Oncogene.

[55] Yugawa K., Itoh S., Yoshizumi T., Iseda N., Tomiyama T., Morinaga A., Toshima T., Harada N., Kohashi K., Oda Y. (2020). CMTM6 Stabilizes PD-L1 Expression and Is a New Prognostic Impact Factor in Hepatocellular Carcinoma. Hepatol. Commun..

[56] McWhorter R., Bonavida B. (2024). The Role of TAMs in the Regulation of Tumor Cell Resistance to Chemotherapy. Crit. Rev. Oncog..

[57] Shree T., Olson O.C., Elie B.T., Kester J.C., Garfall A.L., Simpson K., Bell-McGuinn K.M., Zabor E.C., Brogi E., Joyce J.A. (2011). Macrophages and cathepsin proteases blunt chemotherapeutic response in breast cancer. Genes Dev..

[58] Anderson N.M., Simon M.C. (2020). The tumor microenvironment. Curr. Biol..

[59] Leshner M., Wang S., Lewis C., Zheng H., Chen X.A., Santy L., Wang Y. (2012). PAD4 mediated histone hypercitrullination induces heterochromatin decondensation and chromatin unfolding to form neutrophil extracellular trap-like structures. Front. Immunol..

[60] Arpinati L., Shaul M.E., Kaisar-Iluz N., Mali S., Mahroum S., Fridlender Z.G. (2020). NETosis in cancer: A critical analysis of the impact of cancer on neutrophil extracellular trap (NET) release in lung cancer patients vs. mice. Cancer Immunol. Immunother..

[61] Homa-Mlak I., Majdan A., Mlak R., Małecka-Massalska T. (2016). Metastatic potential of NET in neoplastic disease. Postep. Hig. Med. Dosw..

[62] Masucci M.T., Minopoli M., Del Vecchio S., Carriero M.V. (2020). The Emerging Role of Neutrophil Extracellular Traps (NETs) in Tumor Progression and Metastasis. Front. Immunol..

[63] Fang Q., Stehr A.M., Naschberger E., Knopf J., Herrmann M., Stürzl M. (2022). No NETs no TIME: Crosstalk between neutrophil extracellular traps and the tumor immune microenvironment. Front. Immunol..

[64] Volkov D.V., Tetz G.V., Rubtsov Y.P., Stepanov A.V., Gabibov A.G. (2021). Neutrophil Extracellular Traps (NETs): Opportunities for Targeted Therapy. Acta Naturae.

[65] Darvin P., Toor S.M., Sasidharan Nair V., Elkord E. (2018). Immune checkpoint inhibitors: Recent progress and potential biomarkers. Exp. Mol. Med..

[66] Zhang X., Powell K., Li L. (2020). Breast Cancer Stem Cells: Biomarkers, Identification and Isolation Methods, Regulating Mechanisms, Cellular Origin, and Beyond. Cancers.

[67] Al-Hajj M., Wicha M.S., Benito-Hernandez A., Morrison S.J., Clarke M.F. (2003). Prospective identification of tumorigenic breast cancer cells. Proc. Natl. Acad. Sci. USA.

[68] Ginestier C., Hur M.H., Charafe-Jauffret E., Monville F., Dutcher J., Brown M., Jacquemier J., Viens P., Kleer C.G., Liu S. (2007). ALDH1 is a marker of normal and malignant human mammary stem cells and a predictor of poor clinical outcome. Cell Stem Cell.

[69] Ponti D., Costa A., Zaffaroni N., Pratesi G., Petrangolini G., Coradini D., Pilotti S., Pierotti M.A., Daidone M.G. (2005). Isolation and in vitro propagation of tumorigenic breast cancer cells with stem/progenitor cell properties. Cancer Res..

[70] Chaffer C.L., Marjanovic N.D., Lee T., Bell G., Kleer C.G., Reinhardt F., D’Alessio A.C., Young R.A., Weinberg R.A. (2013). Poised chromatin at the ZEB1 promoter enables breast cancer cell plasticity and enhances tumorigenicity. Cell.

[71] Koren S., Reavie L., Couto J.P., De Silva D., Stadler M.B., Roloff T., Britschgi A., Eichlisberger T., Kohler H., Aina O. (2015). PIK3CA(H1047R) induces multipotency and multi-lineage mammary tumours. Nature.

[72] Morel A.-P., Lièvre M., Thomas C., Hinkal G., Ansieau S., Puisieux A. (2008). Generation of Breast Cancer Stem Cells through Epithelial-Mesenchymal Transition. PLoS ONE.

[73] Velasco-Velázquez M.A., Homsi N., De La Fuente M., Pestell R.G. (2012). Breast cancer stem cells. Int. J. Biochem. Cell Biol..

[74] Crabtree J.S., Miele L. (2018). Breast Cancer Stem Cells. Biomedicines.

[75] D’Angelo R.C., Ouzounova M., Davis A., Choi D., Tchuenkam S.M., Kim G., Luther T., Quraishi A.A., Senbabaoglu Y., Conley S.J. (2015). Notch reporter activity in breast cancer cell lines identifies a subset of cells with stem cell activity. Mol. Cancer Ther..

[76] Grudzien P., Lo S., Albain K.S., Robinson P., Rajan P., Strack P.R., Golde T.E., Miele L., Foreman K.E. (2010). Inhibition of Notch signaling reduces the stem-like population of breast cancer cells and prevents mammosphere formation. Anticancer. Res..

[77] Simmons M.J., Serra R., Hermance N., Kelliher M.A. (2012). NOTCH1 inhibition in vivo results in mammary tumor regression and reduced mammary tumorsphere-forming activity in vitro. Breast Cancer Res..

[78] Takebe N., Miele L., Harris P.J., Jeong W., Bando H., Kahn M., Yang S.X., Ivy S.P. (2015). Targeting Notch, Hedgehog, and Wnt pathways in cancer stem cells: Clinical update. Nat. Rev. Clin. Oncol..

[79] Pannuti A., Foreman K., Rizzo P., Osipo C., Golde T., Osborne B., Miele L. (2010). Targeting Notch to target cancer stem cells. Clin. Cancer Res..

[80] Chung S.S., Vadgama J.V. (2015). Curcumin and epigallocatechin gallate inhibit the cancer stem cell phenotype via down-regulation of STAT3-NFκB signaling. Anticancer Res..

[81] Liu S., Dontu G., Mantle I.D., Patel S., Ahn N.S., Jackson K.W., Suri P., Wicha M.S. (2006). Hedgehog signaling and Bmi-1 regulate self-renewal of normal and malignant human mammary stem cells. Cancer Res..

[82] Fu Y.-Z., Yan Y.-Y., He M., Xiao Q.H., Yao W.F., Zhao L., Wu H.Z., Yu Z.J., Zhou M.Y., Lv M.T. (2016). Salinomycin induces selective cytotoxicity to MCF-7 mammosphere cells through targeting the Hedgehog signaling pathway. Oncol. Rep..

[83] Monteiro J., Gaspar C., Richer W., Franken P.F., Sacchetti A., Joosten R., Idali A., Brandao J., Decraene C., Fodde R. (2014). Cancer stemness in Wnt-driven mammary tumorigenesis. Carcinogenesis.

[84] Jang G.-B., Kim J.-Y., Cho S.-D., Park K.S., Jung J.Y., Lee H.Y., Hong I.S., Nam J.S. (2015). Blockade of Wnt/β-catenin signaling suppresses breast cancer metastasis by inhibiting CSC-like phenotype. Sci. Rep..

[85] Cordenonsi M., Zanconato F., Azzolin L., Forcato M., Rosato A., Frasson C., Inui M., Montagner M., Parenti A.R., Poletti A. (2011). The Hippo transducer TAZ confers cancer stem cell-related traits on breast cancer cells. Cell.

[86] Norton K.A., Wallace T., Pandey N.B., Popel A.S. (2017). An agent-based model of triple-negative breast cancer: The interplay between chemokine receptor CCR5 expression, cancer stem cells, and hypoxia. BMC Syst. Biol..

[87] Chun S.-Y., Kwon Y.-S., Nam K.-S., Kim S. (2015). Lapatinib enhances the cytotoxic effects of doxorubicin in MCF-7 tumorspheres by inhibiting the drug efflux function of ABC transporters. Biomed. Pharmacother..

[88] Singh N., Patel K., Navalkar A., Kadu P., Datta D., Chatterjee D., Mukherjee S., Shaw R., Gahlot N., Shaw A. (2023). Amyloid fibril-based thixotropic hydrogels for modeling of tumor spheroids in vitro. Biomaterials.

[89] Kessel S.L., Chan L.L.-Y. (2020). A High-Throughput Image Cytometry Method for the Formation, Morphometric, and Viability Analysis of Drug-Treated Mammospheres. SLAS Discov..

[90] Lee D., Cha C. (2020). Cell subtype-dependent formation of breast tumor spheroids and their variable responses to chemotherapeutics within microfluidics-generated 3D microgels with tunable mechanics. Mater. Sci. Eng. C Mater. Biol. Appl..

[91] Zhang X., Wang W., Yu W., Xie Y., Zhang X., Zhang Y., Ma X. (2005). Development of an in vitro multicellular tumor spheroid model using microencapsulation and its application in anticancer drug screening and testing. Biotechnol. Prog..

[92] Lemos L.G.T., Longo G.M.d.C., Mendonça B.D.S., Robaina M.C., Brum M.C.M., Cirilo C.D.A., Gimba E.R.P., Costa P.R.R., Buarque C.D., Nestal de Moraes G. (2019). The LQB-223 Compound Modulates Antiapoptotic Proteins and Impairs Breast Cancer Cell Growth and Migration. Int. J. Mol. Sci..

[93] Gilson P., Couvet M., Vanwonterghem L., Henry M., Vollaire J., Baulin V., Werner M., Orlowska A., Josserand V., Mahuteau-Betzer F. (2019). The pyrrolopyrimidine colchicine-binding site agent PP-13 reduces the metastatic dissemination of invasive cancer cells in vitro and in vivo. Biochem. Pharmacol..

[94] Maroufi N.F., Vahedian V., Akbarzadeh M., Mohammadian M., Zahedi M., Isazadeh A., Pouremamali F., Taefehshokr S., Heidari M., Rashidi M. (2020). The apatinib inhibits breast cancer cell line MDA-MB-231 in vitro by inducing apoptosis, cell cycle arrest, and regulating nuclear factor-κB (NF-κB) and mitogen-activated protein kinase (MAPK) signaling pathways. Breast Cancer.

[95] Baker A.E.G., Bahlmann L.C., Tam R.Y., Liu J.C., Ganesh A.N., Mitrousis N., Marcellus R., Spears M., Bartlett J.M., Cescon D.W. (2019). Benchmarking to the Gold Standard: Hyaluronan-Oxime Hydrogels Recapitulate Xenograft Models with In Vitro Breast Cancer Spheroid Culture. Adv. Mater..

[96] Ramanujan V.K. (2019). Quantitative Imaging of Morphometric and Metabolic Signatures Reveals Heterogeneity in Drug Response of Three-Dimensional Mammary Tumor Spheroids. Mol. Imaging Biol..

[97] Di Donato M., Giovannelli P., Migliaccio A., Bilancio A. (2022). Inhibition of Vps34 and p110δ PI3K Impairs Migration, Invasion and Three-Dimensional Spheroid Growth in Breast Cancer Cells. Int. J. Mol. Sci..

[98] Yu Y., Wang Z.-H., Zhang L., Yao H.J., Zhang Y., Li R.J., Ju R.J., Wang X.X., Zhou J., Li N. (2012). Mitochondrial targeting topotecan-loaded liposomes for treating drug-resistant breast cancer and inhibiting invasive metastases of melanoma. Biomaterials.

[99] Li F., Chen W.-L., You B.-G., Liu Y., Yang S.D., Yuan Z.Q., Zhu W.J., Li J.Z., Qu C.X., Zhou Y.J. (2016). Enhanced Cellular Internalization and On-Demand Intracellular Release of Doxorubicin by Stepwise pH-/Reduction-Responsive Nanoparticles. ACS Appl. Mater. Interfaces.

[100] Nahire R., Haldar M.K., Paul S., Ambre A.H., Meghnani V., Layek B., Katti K.S., Gange K.N., Singh J., Sarkar K. (2014). Multifunctional polymersomes for cytosolic delivery of gemcitabine and doxorubicin to cancer cells. Biomaterials.

[101] Meng T., Liu J., Wen L., Yuan M., Cheng B., Hu Y., Zhu Y., Liu X., Yuan H., Hu F. (2016). Multi-cycle chemotherapy with the glycolipid-like polymeric micelles evade cancer stem cell enrichment in breast cancer therapy. Oncotarget.

[102] Stras S., Holleran T., Howe A., Sofou S. (2016). Interstitial Release of Cisplatin from Triggerable Liposomes Enhances Efficacy against Triple Negative Breast Cancer Solid Tumor Analogues. Mol. Pharm..

[103] Muddineti O.S., Vanaparthi A., Rompicharla S.V.K., Kumari P., Ghosh B., Biswas S. (2018). Cholesterol and vitamin E-conjugated PEGylated polymeric micelles for efficient delivery and enhanced anticancer activity of curcumin: Evaluation in 2D monolayers and 3D spheroids. Artif. Cells Nanomed. Biotechnol..

[104] Ciardelli G., Boccaccini A.R., Roy I., Nostro A., Salber J. (2021). Editorial: Combating Bacterial Infections Through Biomimetic or Bioinspired Materials Design and Enabling Technologies. Front. Bioeng. Biotechnol..

[105] Brancato V., Gioiella F., Imparato G., Guarnieri D., Urciuolo F., Netti P.A. (2018). 3D breast cancer microtissue reveals the role of tumor microenvironment on the transport and efficacy of free-doxorubicin in vitro. Acta Biomater..

[106] Ehsan S.M., Welch-Reardon K.M., Waterman M.L., Hughes C.C., George S.C. (2014). A three-dimensional in vitro model of tumor cell intravasation. Integr. Biol..

[107] Hsieh P.H., Huang W.Y., Wang H.C., Kuan C.H., Shiue T.Y., Chen Y., Wang T.W. (2022). Dual-responsive polypeptide nanoparticles attenuate tumor-associated stromal desmoplasia and anticancer through programmable dissociation. Biomaterials.

[108] Salerno D., Sofou S. (2021). Growth Inhibition of Triple-Negative Breast Cancer: The Role of Spatiotemporal Delivery of Neoadjuvant Doxorubicin and Cisplatin. Pharmaceuticals.

[109] Chou H.-H., Che T.-F., Lee K.-J., Chen S.C., Chen J.Y., Huang Y.J., Lim S.C., Huang S.C., Tsai C.L., Chang Y.C. (2026). Application of CTC-derived spheroid for drug screening toward personalized treatment in patients with breast cancer. Transl. Oncol..

[110] Piwocka O., Sterzyńska K., Malińska A., Suchorska W.M., Kulcenty K. (2025). Development of tetraculture spheroids as a versatile 3D model for personalized breast cancer research. Sci. Rep..

[111] Ascheid D., Baumann M., Pinnecker J., Friedrich M., Szi-Marton D., Medved C., Bundalo M., Ortmann V., Öztürk A., Nandigama R. (2024). A vascularized breast cancer spheroid platform for the ranked evaluation of tumor microenvironment-targeted drugs by light sheet fluorescence microscopy. Nat. Commun..

[112] Dontu G., Abdallah W.M., Foley J.M., Jackson K.W., Clarke M.F., Kawamura M.J., Wicha M.S. (2003). In vitro propagation and transcriptional profiling of human mammary stem/progenitor cells. Genes Dev..

[113] Jiao X., Rizvanov A.A., Cristofanilli M., Miftakhova R.R., Pestell R.G. (2016). Breast Cancer Stem Cell Isolation. Methods Mol. Biol..

[114] Shaw F.L., Harrison H., Spence K., Ablett M.P., Simões B.M., Farnie G., Clarke R.B. (2012). A detailed mammosphere assay protocol for the quantification of breast stem cell activity. J. Mammary Gland. Biol. Neoplasia.

[115] Charafe-Jauffret E., Ginestier C., Iovino F., Wicinski J., Cervera N., Finetti P., Hur M.H., Diebel M.E., Monville F., Dutcher J. (2009). Breast Cancer Cell Lines Contain Functional Cancer Stem Cells with Metastatic Capacity and a Distinct Molecular Signature. Cancer Res..

[116] Li Y., Zhang T., Korkaya H., Liu S., Lee H.F., Newman B., Yu Y., Clouthier S.G., Schwartz S.J., Wicha M.S. (2010). Sulforaphane, a Dietary Component of Broccoli/Broccoli Sprouts, Inhibits Breast Cancer Stem Cells. Clin. Cancer Res..

[117] Lamb R., Harrison H., Smith D.L., Townsend P.A., Jackson T., Ozsvari B., Martinez-Outschoorn U.E., Pestell R.G., Howell A., Lisanti M.P. (2015). Targeting tumor-initiating cells: Eliminating anabolic cancer stem cells with inhibitors of protein synthesis or by mimicking caloric restriction. Oncotarget.

[118] Wanandi S.I., Limanto A., Yunita E., Syahrani R.A., Louisa M., Wibowo A.E., Arumsari S. (2020). In silico and in vitro studies on the anti-cancer activity of andrographolide targeting survivin in human breast cancer stem cells. PLoS ONE.

[119] Choi H.S., Kim S.-L., Kim J.-H., Deng H.Y., Yun B.S., Lee D.S. (2018). Triterpene Acid (3-O-p-Coumaroyltormentic Acid) Isolated from Aronia Extracts Inhibits Breast Cancer Stem Cell Formation through Downregulation of c-Myc Protein. Int. J. Mol. Sci..

[120] Yang Y., Hao E., Pan X., Tan D., Du Z., Xie J., Hou X., Deng J., Wei K. (2019). Gomisin M2 from Baizuan suppresses breast cancer stem cell proliferation in a zebrafish xenograft model. Aging.

[121] Scatena C., Roncella M., Paolo A.D., Aretini P., Menicagli M., Fanelli G., Marini C., Mazzanti C.M., Ghilli M., Sotgia F. (2018). Doxycycline, an Inhibitor of Mitochondrial Biogenesis, Effectively Reduces Cancer Stem Cells (CSCs) in Early Breast Cancer Patients: A Clinical Pilot Study. Front. Oncol..

[122] Safi R., Hamade A., Bteich N., El Saghir J., Assaf M.D., El-Sabban M., Najjar F. (2018). A ferutinin analogue with enhanced potency and selectivity against ER-positive breast cancer cells in vitro. Biomed. Pharmacother..

[123] Zhou Z., Feng Z., Hu D., Yang P., Gur M., Bahar I., Cristofanilli M., Gradishar W.J., Xie X.Q., Wan Y. (2019). A novel small-molecule antagonizes PRMT5-mediated KLF4 methylation for targeted therapy. eBioMedicine.

[124] Kim D.-A., Choi H.S., Ryu E.-S., Ko J., Shin H.S., Lee J.M., Chung H., Jun E., Oh E.S., Kang D.H. (2019). Tannic acid attenuates the formation of cancer stem cells by inhibiting NF-κB-mediated phenotype transition of breast cancer cells. Am. J. Cancer Res..

[125] Zhang X., Li F., Zheng Y., Wang X., Wang K., Yu Y., Zhao H. (2019). Propofol Reduced Mammosphere Formation of Breast Cancer Stem Cells via PD-L1/Nanog In Vitro. Oxidative Med. Cell. Longev..

[126] Sharma A., Paranjape A.N., Rangarajan A., Dighe R.R. (2012). A monoclonal antibody against human Notch1 ligand-binding domain depletes subpopulation of putative breast cancer stem-like cells. Mol. Cancer Ther..

[127] Geller M.A., Cooley S., Judson P.L., Ghebre R., Carson L.F., Argenta P.A., Jonson A.L., Panoskaltsis-Mortari A., Curtsinger J., McKenna D. (2010). A phase II study of allogeneic natural killer cell therapy to treat patients with recurrent ovarian and breast cancer. Cytotherapy.

[128] Byrd T.T., Fousek K., Pignata A., Szot C., Samaha H., Seaman S., Dobrolecki L., Salsman V.S., Oo H.Z., Bielamowicz K. (2017). TEM8/ANTXR1-specific CAR T cells as a targeted therapy for triple-negative breast cancer. Cancer Res..

[129] Li S.-Y., Sun R., Wang H.-X., Shen S., Liu Y., Du X.J., Zhu Y.H., Jun W. (2015). Combination therapy with epigenetic-targeted and chemotherapeutic drugs delivered by nanoparticles to enhance the chemotherapy response and overcome resistance by breast cancer stem cells. J. Control. Release.

[130] Desai A., Yan Y., Gerson S.L. (2018). Concise Reviews: Cancer Stem Cell Targeted Therapies: Toward Clinical Success. Stem Cells Transl. Med..

[131] Chu X., Tian W., Ning J., Xiao G., Zhou Y., Wang Z., Zhai Z., Tanzhu G., Yang J., Zhou R. (2024). Cancer stem cells: Advances in knowledge and implications for cancer therapy. Signal Transduct. Target. Ther..

[132] Zhang F. Medicine’s New Frontier. University of Miami Medicine Magazine. https://magazine2.med.miami.edu/medicines-new-frontier/.

[133] Goetz L.H., Schork N.J. (2018). Personalized Medicine: Motivation, Challenges and Progress. Fertil. Steril..

[134] CDC The Shift from Personalized Medicine to Precision Medicine Precision Public Health: Words Matter! |Blogs|. https://blogs.cdc.gov/genomics/2016/04/21/shift/.

[135] Subhan M.A., Parveen F., Shah H., Yalamarty S.S.K., Ataide J.A., Torchilin V.P. (2023). Recent Advances with Precision Medicine Treatment for Breast Cancer including Triple-Negative Sub-Type. Cancers.

[136] Jitariu A.-A., Cîmpean A.M., Ribatti D., Raica M. (2017). Triple negative breast cancer: The kiss of death. Oncotarget.

[137] Phung M.T., Tin Tin S., Elwood J.M. (2019). Prognostic models for breast cancer: A systematic review. BMC Cancer.

[138] Ma D., Hernandez G.A., Lefebvre A.E.Y.T., Alshetaiwi H., Blake K., Dave K.R., Rauf M., Williams J.W., Davis R.T., Evans K.T. (2021). Patient-derived xenograft culture-transplant system for investigation of human breast cancer metastasis. Commun. Biol..

[139] Papaccio F., Cabeza-Segura M., Garcia-Micò B., Tarazona N., Roda D., Castillo J., Cervantes A. (2022). Will Organoids Fill the Gap towards Functional Precision Medicine?. J. Pers. Med..

[140] Gilazieva Z., Ponomarev A., Rutland C., Rizvanov A., Solovyeva V. (2020). Promising Applications of Tumor Spheroids and Organoids for Personalized Medicine. Cancers.

[141] Halfter K., Ditsch N., Kolberg H.-C., Fischer H., Hauzenberger T., von Koch F.E., Bauerfeind I., von Minckwitz G., Funke I., Crispin A. (2015). Prospective cohort study using the breast cancer spheroid model as a predictor for response to neoadjuvant therapy—The SpheroNEO study. BMC Cancer.

[142] Hofmann S., Cohen-Harazi R., Maizels Y., Koman I. (2022). Patient-derived tumor spheroid cultures as a promising tool to assist personalized therapeutic decisions in breast cancer. Transl. Cancer Res..

[143] Halfter K., Hoffmann O., Ditsch N., Ahne M., Arnold F., Paepke S., Grab D., Bauerfeind I., Mayer B. (2016). Testing chemotherapy efficacy in HER2 negative breast cancer using patient-derived spheroids. J. Transl. Med..

[144] Chen X., Sifakis E.G., Robertson S., Neo S.Y., Jun S.H., Tong L., Hui Min A.T., Lövrot J., Hellgren R., Margolin S. (2023). Breast cancer patient-derived whole-tumor cell culture model for efficient drug profiling and treatment response prediction. Proc. Natl. Acad. Sci. USA.

[145] Peterziel H., Jamaladdin N., ElHarouni D., Gerloff X.F., Herter S., Fiesel P., Berker Y., Blattner-Johnson M., Schramm K., Jones B.C. (2022). Drug sensitivity profiling of 3D tumor tissue cultures in the pediatric precision oncology program INFORM. npj Precis. Oncol..

[146] Shuford S., Wilhelm C., Rayner M., Elrod A., Millard M., Mattingly C., Lotstein A., Smith A.M., Guo Q.J., O’Donnell L. (2019). Prospective Validation of an Ex Vivo, Patient-Derived 3D Spheroid Model for Response Predictions in Newly Diagnosed Ovarian Cancer. Sci. Rep..

[147] Crouchet E., Almeida N., Durand S.C., Parnot M., Oudot M.A., Giannone F., Gadenne C., Roehlen N., Saviano A., Felli E. (2024). A patient-derived HCC spheroid system to model the tumor microenvironment and treatment response. JHEP Rep..

[148] Mehta G., Hsiao A.Y., Ingram M., Luker G.D., Takayama S. (2012). Opportunities and challenges for use of tumor spheroids as models to test drug delivery and efficacy. J. Control. Release.

[149] Cordeiro S., Oliveira B.B., Valente R., Ferreira D., Luz A., Baptista P.V., Fernandes A.R. (2024). Breaking the mold: 3D cell cultures reshaping the future of cancer research. Front. Cell Dev. Biol..

[150] Arora S., Singh S., Mittal A., Desai N., Khatri D.K., Gugulothu D., Lather V., Pandita D., Vora L.K. (2024). Spheroids in cancer research: Recent advances and opportunities. J. Drug Deliv. Sci. Technol..

[151] Han S.J., Kwon S., Kim K.S. (2021). Challenges of applying multicellular tumor spheroids in preclinical phase. Cancer Cell Int..

[152] Zhu S., Yin J., Lu X., Jiang D., Chen R., Cui K., He W., Huang N., Xu G. (2025). Influence of experimental variables on spheroid attributes. Sci. Rep..

[153] Barisam M., Saidi M.S., Kashaninejad N., Nguyen N.T. (2018). Prediction of Necrotic Core and Hypoxic Zone of Multicellular Spheroids in a Microbioreactor with a U-Shaped Barrier. Micromachines.

[154] Singh S.K., Abbas S., Saxena A.K., Tiwari S., Sharma L.K., Tiwari M. (2020). Critical role of three-dimensional tumorsphere size on experimental outcome. BioTechniques.

[155] Tevlek A., Kecili S., Ozcelik O.S., Kulah H., Tekin H.C. (2023). Spheroid Engineering in Microfluidic Devices. ACS Omega.

[156] Lee S., Kim N., Kim S.H., Um S.J., Park J.Y. (2023). Biological and mechanical influence of three-dimensional microenvironment formed in microwell on multicellular spheroids composed of heterogeneous hair follicle stem cells. Sci. Rep..

[157] Shi Y., Guan Z., Cai G., Nie Y., Zhang C., Luo W., Liu J. (2024). Patient-derived organoids: A promising tool for breast cancer research. Front. Oncol..

[158] Liu M., Yang X. (2025). Patient-derived xenograft models: Current status, challenges, and innovations in cancer research. Genes Dis..

[159] Tosca E.M., Ronchi D., Facciolo D., Magni P. (2023). Replacement, Reduction, and Refinement of Animal Experiments in Anticancer Drug Development: The Contribution of 3D In Vitro Cancer Models in the Drug Efficacy Assessment. Biomedicines.

[160] Leung D., Kaur J., Richardson G., Jardé T. (2025). Breast cancer organoids: Advancements and applications in precision medicine. Crit. Rev. Oncol. Hematol..

[161] Shannon A.E., Boos C.E., Hummon A.B. (2021). Co-culturing multicellular tumor models: Modeling the tumor microenvironment and analysis techniques. Proteomics.

[162] Rossi M., Blasi P. (2022). Multicellular Tumor Spheroids in Nanomedicine Research: A Perspective. Front. Med. Technol..

[163] Lee K.H., Kim T.H. (2021). Recent Advances in Multicellular Tumor Spheroid Generation for Drug Screening. Biosensors.

[164] Guillen K.P., Fujita M., Butterfield A.J., Scherer S.D., Bailey M.H., Chu Z., DeRose Y.S., Zhao L., Cortes-Sanchez E., Yang C.H. (2022). A human breast cancer-derived xenograft and organoid platform for drug discovery and precision oncology. Nat. Cancer.

[165] Hou X., Du C., Lu L., Yuan S., Zhan M., You P., Du H. (2022). -Opportunities and challenges of patient-derived models in cancer research: Patient-derived xenografts, patient-derived organoid and patient-derived cells. World J. Surg. Oncol..

